# Identifying the impact of rainfall variability on conflicts at the monthly level

**DOI:** 10.1038/s41598-022-23079-y

**Published:** 2022-10-28

**Authors:** Thierry Yerema Coulibaly, Shunsuke Managi

**Affiliations:** Urban Institute & Department of Civil Engineering, Kyushu University, 744 Motooka, Nishi-Ku, Fukuoka, 819-0395 Japan

**Keywords:** Environmental impact, Governance, Risk factors

## Abstract

Research on the relationship between rainfall variability and conflicts has yielded contradictory results. This study is the first to show that the significance of the impact of rainfall variability on conflicts depends on the temporal unit of analysis. We prove this point by comparing the statistical significance of the linkages between georeferenced conflicts and rainfall variabilities at the monthly and annual levels with panel data analyses from 1989 to 2020. We find that a 10 percent increase in monthly rainfall decreases the risk of conflict incidence by 0.0298 percent, but annual rainfall variability is not statistically linked to conflict outbreaks. These statistically significant disparities result from the aggregation of data dispersion and the disregard for the timing of the impact of rainfall on conflicts. These findings highlight the importance of information on monthly rainfall variation when estimating the impact of rainfall on conflicts.

## Introduction

Societies continue to be plagued by armed conflicts. These conflicts that can be defined as violent confrontations during which there is a use of armed force caused 101,400 fatalities in 2014, making it the most violent year in the post-Cold War period^1^. Historically, low socioeconomic development, state capabilities, and intergroup inequality stand out as the most significant drivers of armed conflicts^2^. However, recent evidence shows that climate anomalies may also contribute to conflict outbreaks.

Studies in psychology and economics demonstrate that individuals exhibit violent behavior when affected by extreme climatic conditions^3^. For instance, extreme rainfall variations were shown to increase personal violence in the case of witch killings in Tanzania^4^, property crime in Germany^5^, and armed conflicts in India^6^. Nevertheless, there is a scholarly debate over the generalization of these phenomena at the macro-level and large-scale violence, such as armed conflicts worldwide.

Table 1 summarizes a sample of studies assessing the link between rainfall and conflicts. It contrasts these studies relative to their support or disapproval of a statistically significant relationship between these two variables. On the one hand, it was repeatedly found that low water availability increases the risk of conflicts^3,7–10^. For instance, most pessimistic estimates in Africa suggest that negative rainfall anomalies relative to long-term average values increased the risk of armed conflict by 45 percent^11^.

**Table 1:** Non-exhaustive list of empirical studies assessing the relationship between rainfall factors and conflict.

Author	Dependent variable	Independent variable	Sample region	Sample period	Time unit	Spatial unit	Method for temporally aggregating rainfall at the yearly level	Statistical significance	Refs.
**Studies reporting no statistically significant relationship between rainfall and conflicts**									
Theisen et al. (2012)	Civil war onset	Rainfall and drought	Africa	1960–2004	Year	Pixel (0.5^0^)	SPI6 index measuring monthly negative deviation from normal rainfall during the six earlier months is averaged yearly	–	^20^
van Weezel et al. (2015)	conflict onset	Rainfall (IV economic growth)	Africa	1981–2010	Year	Country	Arithmetic average	–	^21^
Buhaug et al. (2010)	Civil conflict	Rainfall deviation	Africa	1960–2004	Year	Country	Arithmetic average	–	^17^
Wischnath et al. (2014)	Civil conflict onset	Rainfall (IV economic growth)	Asia	1950–2008	Year	Pixel (0.5^0^)	Arithmetic average	–	^26^
Ciccone et al. (2011)	Civil conflict incidence and onset	Rainfall (IV economic growth)	41 African countries	1979–2009	Year	Country	Arithmetic average	–	^27^
Bergholt et al. (2012)	Civil conflict onset	Flood (IV economic growth)	171 countries worldwide	1980–2007	Year	Country	Normalized values of disaster by month of incidence and population affected	–	^28^
Koubi et al. (2012)	Civil conflict onset	Rainfall (IV for economic growth)	Global and Africa	1980–2004	Year	Country	Arithmetic average	–	^29^
Buhaug, et al. (2015)	civil conflict onset	Rainfall (IV agricultural yields)	Sub-Saharan Africa	1962–2009	Year	Country	Arithmetic average	–	^30^
Burke et al. (2009)	Civil war incidence	Precipitation	Sub-Saharan Africa	1981–2012	Year	Country	Arithmetic average	–	^23^
von Uexkull et al. (2016)	Communal conflict	Growing season drought	Africa and Asia	1989–2014	Year	Ethnic group	Monthly rainfalls are weighted relative to the growing seasons of the primary crops and averaged per year	–	^42^
Burke et al. (2009)	Civil war incidence	Rainfall	Sub-Saharan Africa	1981–2002	Year	Country	Arithmetic average	–	^23^
Harari et al. (2018)	Conflict incidence	Rainfall (see Table A10 in their analysis)	Africa	1960–2010	Year	Pixel (0.1^0^)	Sum over the growing season	–	^24^
**Studies reporting a statistically significant relationship between rainfall and conflicts**									
O’Loughlin et al., (2012)	Civil war and social conflict	Precipitation anomalies	Horn and Eastern Africa	1990–2009	Month	Pixel (0.1^0^)	Sum of rain over six months standardized over the long-term mean	**	^48^
Fjelde et al., (2012)	Communal conflict	Rainfall	Sub-Saharan Africa	1990–2008	Year	Province	Arithmetic average	*	^11^
McGuirk et al. (2020)	State and non-state conflicts	Rainfall (interacted with transhuman pastoralism)	Africa	1989–2018	Year and Month	Ethnic groups and pixel (0.5^0^)	Arithmetic average	***	^16^
Caruso et al. (2016)	Number of violent incidents	Rainfall (IV of agricultural production)	Indonesia	1993–2003	Month	Province	None	**	^32^
Raleigh et al. (2012)	Civil wars and communal conflicts	Rainfall	Uganda, Kenya, and Ethiopia	1997–2009	Month	Conflict location	None	***	^9^
Hendrix et al. (2012)	Civil conflict and social conflict	Rainfall	Africa	1998–2008	Year	Country	Arithmetic average	***	^7^
Smith et al. (2014)	Urban sociopolitical unrest	Rainfall (IV for food price shocks)	Africa	1990–2012	Month	Country	None	***	^34^
Raleigh et al. (2015)	Violent conflict event	Rainfall/ drought and rainy season (IV for commodity price)	Africa	1997–2010	Month	Country	Administrative regions	**	^35^
Hodler et al. (2014)	Civil conflict incidence	Rainfall and Palmer Drought Index (IV for nighttime lights)	Africa	1992–2010	Year	Regions	Arithmetic average	***	^12^
Bohlken et al. (2010)	Ethnic riots	Rainfall (IV for economic growth)	India	1982–1995	Year	Provinces	Arithmetic average	***	^47^
Blakelslee et al. (2017)	Crime incidence	Rainfall (IV for agricultural income)	India	1971–2000	Year and seasons	District	Arithmetic average	***	^6^
Sarsons et al. (2015)	Ethnic riots	Rainfall shocks	India	1970–1995	Year	District	Sum of monthly rainfall deviations from the historical average	***	^10^
Crost et al. (2017)	Number of conflicts	Rainfall (IV for agricultural production)	Philippines	2001–2009	Year and seasons	Province	Arithmetic average per season/year	- (for yearly estimates of rainfall) and *** (for seasonal rainfall)	^33^
Hidalgo et al. (2010)	Land invasion	Rainfall (IV agricultural income)	Brazil	1988–2004	Year and month	Municipality	Sum of monthly rainfall standard values	***	^13^
David Helman et al. (2020)	Armed conflicts incidence	Standardized rainfall	Africa and the Middle East	1992–2012	Year	Pixel (0.5^0^)	Arithmetic average	***	^43^

The column titled Ref. stands for References. The symbol in the column displaying the statistical significance of the linkages between rainfall and conflicts conveys the following ***p < 0.01, **p < 0.05, *p < 0.1, − p > 0.1). IV stands for the instrumental variable. The column titled independent variable only reports rainfall-related variables even if other variables are included in the original study referenced. The studies of this table stem from previous reviews of the literature^3,8,25^ and are complemented by the author via queries on google scholar. We only report studies that used an econometrics analysis. We limit this list of studies to publications since 2007 (in the past 15 years) as the reporting of the total body of work on this topic is out of the scope of the current analysis.

Previous studies show that these conflicts tend to concern intergroup violence over access to scarce resources resulting from lower rainfall availability^11,14^. For instance, major rainfall deviations may lower income which is theorized to be a major predictor of conflicts. These rainfall deviations may also cause disagreement over the allocation of limited resources, or they may shape the appeal to the use of violence for a preconceived objective^3^. More explicitly, rainfall variability can cause clashes between migrants seeking new means of life unaffected by rainfall deviations and autochthone populations welcoming these migrants to their lands with limited resources^15^. Rainfall deviations may also cause conflicts due to limited access to communal wells or grazelands^11,16^.

On the other hand, Table 1 shows that numerous studies fail to find a statistically significant link between conflicts and rainfall^17–24^. According to surveys of the literature, divergences in model specifications, variables used to explain rainfall loss, data sources, and study areas are the main reasons for discrepancies between the results of previous works^3,18^. However, Table 1 reveals that besides these reasons, studies reporting no statistically significant rainfall-conflict linkage have one common feature: they use an annual rainfall index in their analyses^3,18,20,21,25–30^. Since rainfall variation may exacerbate conflicts through mechanisms that operate at the monthly level, yearly level analyses may obscure the relationship between rainfall and conflicts.

Therefore, this study hypothesizes that difficulties in finding a link between rainfall variability and conflict incidence are due to the lack of attention paid to the temporal unit of analysis. For this purpose, the analysis assesses the effect of climate variability, measured by yearly and monthly rainfall variations, on conflict outbreaks. The scope of studies on climate variability is mostly restricted to these temporal units (year and month) because decadal and centurial variations in precipitation relate to climate change, and daily or hourly variations in precipitation refer to weather variation.

Regarding rainfall variability, numerous pieces of evidence highlight the detrimental impact of rainfall on conflicts dictated by intra-yearly rainfall variations. For instance, conflicts linked to seasonal migrations of transhumant pastoralists for animal grazing^14,16,31^, rainfall loss during the growing seasons of crops^32,33^, increase in food prices^34,35^, and food insecurity^36^ are highly influenced by rainfall changes at the monthly and seasonal levels. In addition, macroeconomic studies investigating the event coincidence between natural disasters, like rainfall shocks, find a correlation between disasters and conflicts only when the periods of analysis do not exceed three months^37^. These effects of rainfall variations on conflicts may be undiscernible for yearly averages. In other words, hydrological studies show that even when annual rainfall does not change significantly, rainy seasons can be wetter while dry seasons can be drier^38,39^. In these instances, populations may be affected by varying monthly rainfall levels, and their reactions to these variations may result in conflicts.

Consequently, this study investigates the hypotheses that there is a statistically significant relationship between precipitation variation and conflict outbreaks at the monthly level but not at the yearly level at subnational administrative levels. Therefore, the effects of rainfall on conflict incidences when rainfall is measured at the monthly and yearly levels are compared. Analyses are performed with georeferenced armed conflict data provided by the Uppsala conflict data program georeferenced event dataset (UCDP GED) from 1989 to 2020 in Africa, Asia, and the Americas^40^. UCDP GED defines armed conflicts as all battle-related events that resulted in at least 25 fatalities. Although most research focuses on Africa, the study uses the UCDP GED dataset covering three continents to provide global interpretations on this topic^41^. This analysis only considers these continents because they exhibit a number of conflict outbreaks over the period of study that can warrant a statistical analysis (see Fig. S1 in Supplementary Information (SI)). Europe and Oceania only experienced 15 and 7 conflict incidences, respectively, between 1989 and 2020. Furthermore, the study focuses on non-state conflicts at the subnational level, which is consistent with previous research indicating that rainfall-induced stress on conflict risks is more likely to affect non-state actors such as citizens^11,42,43^. These conflicts relate to cattle-raiding, pastoralists, farmers, herders’ violence, clashes between militias, or attacks on civilians. Further analyses with conflicts involving state actors are also performed.

Then, monthly and yearly historical data is collected from CHRIPS^44^. This dataset provides estimations of precipitation worldwide at a 0.05° resolution. After averaging these rainfall estimates at the first order of national administrative units, which henceforth are referred to simply as “region”, rainfall is merged with conflict data. These regions suit the current investigation as Fig. S2 in SI shows that regions’ boundaries shape conflict incidence worldwide. These regions capture group-level dynamics, which can serve as causes of conflicts across populations, and which depend on precipitation levels. These group dynamics can be related to the structure of the local economies based on farming, pastoralism, political processes, electoral contests, or the provision of public goods^11^. Analyses do not use countries and ethnic group settlements as main units of observation because country-level analyses are insufficiently precise, and data on ethnic settlements are primarily relevant to Africa. Alternatively, grid pixels as observation units would fail to capture group-level dynamics across populations.

This study uses panel data with a fixed effects model regressed using an Ordinary Least Squares (OLS) estimator to investigate the impact of rainfall on conflict incidence and onset. OLS is used to provide easily understandable estimates of the fixed effects and to compare several model alternatives. As robustness checks, logit and multilevel mixed effects regressions are employed to determine if the OLS is biased due to the binary character of conflict incidence.

The principal analysis does not include control variables because they may capture parts of the total impact of rainfall on armed conflicts. There is a scholarly consensus that the relationship between climate and conflict is indirect or conditional on many factors such as income and population^2^. Current progress focuses on identifying the numerous factors that are causally affected by rainfall and that increase conflict risks due to this causal impact. Thus, regressions including proxy of these factors as control variables will yield estimates of the impact of rainfall when these factors are constant. These eventual regressions would, thereby, inform on a partial impact of climate on conflicts. Our regressions rely on the exogenous nature of rainfall variations to estimate its total impact (see “Methods” for further details on the identification strategy). Control variables are only considered in robustness checks.

Nevertheless, the primary analyses flexibly control for global contemporaneous shocks, region-specific features fixed in time, number of years in peace, and eventual correlation between regions within the country. This fixed effect regression technique allows the comparison of each region with itself when affected by different levels of precipitation^3^. It provides credible estimates of the average causal impact of rainfall on conflicts since rainfall variations are exogenous.

## Results

Table 2 reports the average relationship between rainfall variability and conflicts (incidence and onset) when data are averaged monthly and yearly. Positive monthly rainfall variability decreases the risk of conflict incidence (p = 0.048) but not onset. In the preferred model specification (Model 1, Table 2), an extra millimeter (mm) of regional monthly rainfall relative to long-term average rainfall decreases the risk of conflict incidence by 0.00298%. This 1 mm corresponds to approximately one percentage increase in rainfall relative to the global average (103.2 mm/month, see Table S3). In other words, on average, a 10 percent increase in monthly rainfall results in 0.0298 percent less conflict risk. Although statistically significant, the small magnitude of this estimate shows that rainfall is a minor driver of conflicts and is not statistically related to new conflicts. However, performing the same regression with data averaged annually produces estimates that are not statistically significantly different from 0 at the 10% statistical confidence for conflict incidence and onset (Table 2).

**Table 2:** Comparison of the estimate between the effects of the monthly and yearly average of rainfall on conflicts.

Variables	Model 1—Observations averaged at the monthly level		Model 1—Observations averaged at the annual level	
	Incidence	Onset	Incidence	Onset
Rainfall (mm)	− 2.98e−06** (1.40e−06)	− 8.33e−07 (6.13e−07)	− 3.01e−06 (3.42e−06)	− 1.62e−07 (1.89e−06)
Number of years without conflict (t − 1)	− 0.00433*** (0.00119)	0.000256*** (3.02e−05)	− 0.00614*** (0.00185)	0.00303*** (0.000315)
Constant	0.0699*** (0.0171)	− 0.00269*** (0.000463)	0.125*** (0.0287)	− 0.0339*** (0.00530)
Observations	982,778	923,760	82,112	79,546
R-squared	0.150	0.008	0.288	0.080
Time FE	YES	YES	YES	YES
Region FE	YES	YES	YES	YES
Period	1990–2020	1991–2020	1990–2020	1991–2020
Sample	Full sample	Full sample	Full sample	Full sample

Standard errors are clustered at the country level.

***p < 0.01, **p < 0.05, *p < 0.1.

**Figure 1:**
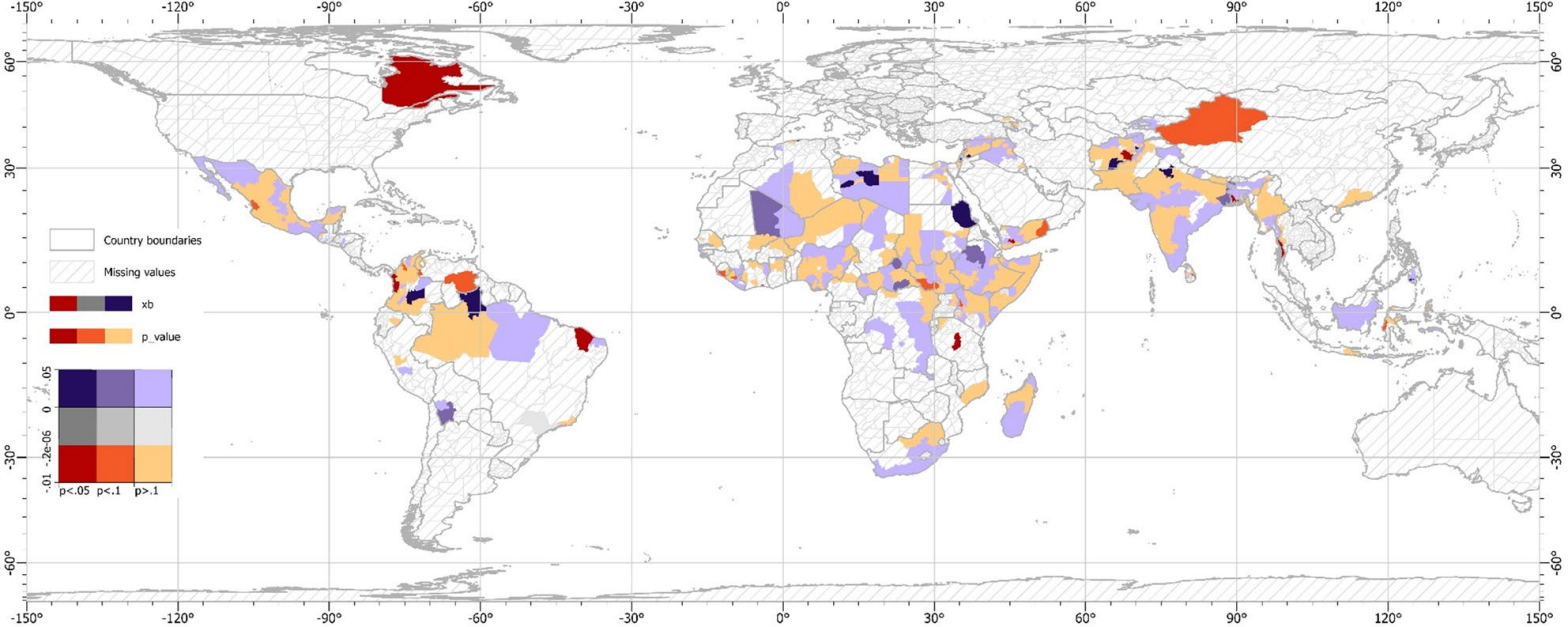
Estimated impact of rainfall on conflict by regions per year-month. This map uses a bivariate color scheme to display rainfall's effect on conflict incidence. This effect is embodied by the coefficient estimate of a regression analysis and the p-value of this coefficient. A separate regression is run for each region (see Supplementary Information). The graph excludes regions that did not experience any conflict between 1989 and 2020 and regions whose regression does not yield a coefficient estimate with a corresponding statistical significance. The map is projected using a cylindric equal area projection. The value of − 2e−06 in the coefficient estimates refers to the average impact of rainfall on conflicts in Table 1. These maps were created using ArcGIS Pro 2.8 (https://www.esrij.com/products/arcgis-pro/).

The divergence of results between monthly and yearly analyses is not heterogeneous based on the income levels of regions and countries. The results are comparable across low- and high-income regions within countries, as well as low- and high-income countries (Table S1). Thus, the divergence between yearly and monthly analyses is not influenced by the inclusion of high-income countries or regions in our sample. Similarly, regional-specific analyses show that the impact of increases in rainfall on conflicts is scattered across regions of all continents considered in the analysis (see Fig. 1). Thus, the impact of rainfall is not restricted to a certain group of countries or regions in the world. However, certain regions experience a higher risk of conflict when faced with higher precipitation. Previous studies have highlighted this positive relationship in Sub-Saharan Africa at the monthly level^45^. It demonstrates that, while rainfall reduces the risk of conflict, the relationship varies depending on the context and region.

This divergence of result with respect to the temporal unit of observations is robust to most alternative model specifications regarding conflict incidence. Monthly level analyses show that higher rainfall reduces conflict incidence when excluding the time fixed effects (SI Table S4, Model 3), regions fixed effects (SI Table S4, Model 4), or including regional income, life expectancy, and total population as control variables (SI Table S4, Model 5). In other words, the impact of monthly rainfall on conflict risks is not affected by (1) time-trending global patterns in climate, population, economic growth, or reporting of conflict data; (2) it is not driven by comparisons across regions rather than within regions at different moments in time; and (3) it does not vary because of differences of regional socioeconomic factors. Moreover, altering the sample of analysis mainly supports the previous findings. This is shown by limiting the sample of regions to those that experienced at least one conflict during the period of study or to African regions, on which most prior studies focus (SI Table S4, Model 6, and Model 7). Except for the sample restriction to African regions, Models 4 to 7 yield no link between rainfall and conflicts when analyses are carried out at the yearly level (SI Table S5). However, one notes that rainfall has a statistically significant impact on conflict incidence (p = 0.09) and onset (p = 0.04) in Africa at the yearly level.

These findings revealing that rainfall affect conflict at the monthly level but not at the yearly level are also robust to using different data and regression techniques (SI Tables S6 and S7). Using the number of conflicts in a month as the dependent variable instead of a binary variable of conflict occurrence reveals that an extra mm of rainfall is linked to a 0.00133 lower number of conflicts (p < 0.01, SI Table S6 Model 8). Moreover, monthly rainfall observations’ impact on conflicts persists when using a logit estimation technique or a two-level mixed effects linear regression with random intercepts at the country level and control variable at the regional level (SI Table S6, Models 9 and 10, respectively). Regressions with the spatial autoregressive model robust to spatial correlation (SI Table S6, Model 11) and block bootstrapping the standard errors to correct for potential serial auto-correlation (SI Table S6, Model 12) also confirm the results.

Furthermore, outlier observations do not drive these results (SI Tables S10 and S11). Measuring rainfall as standardized values with respect to long-term regional average or as logarithm support previous interpretations (Model 13 and Model 15, respectively). Supplementary analyses that restrict the sample to standardized rainfall values between thresholds of − 3 and 3 to drop outliers also confirm previous results (Model 14).

Altering the definition of conflicts bolsters the results suggesting that monthly level regressions better capture the impact of rainfall on conflicts (SI Tables S12 and S13). Considering conflicts involving states (Model 16) or armed clashes as defined by the ACLED data (Model 17) confirms previous estimates. Finally, investigations at the country level rather than the regional level are in line with previous interpretations (Model 18).

Finally, since some robustness checks require different sample specifications, we ensure that differences in samples do not affect the confidence in the results. Previous regressions are performed with regions (1) that experience at least one conflict (see Fig. S1) and (2) where all control variables are available between 1991 and 2019 (Fig. S3). This smaller number of regions provides a sample consistent across regressions that mostly validate previous estimations (see SI Tables S14 to S21).

**Figure 2:**
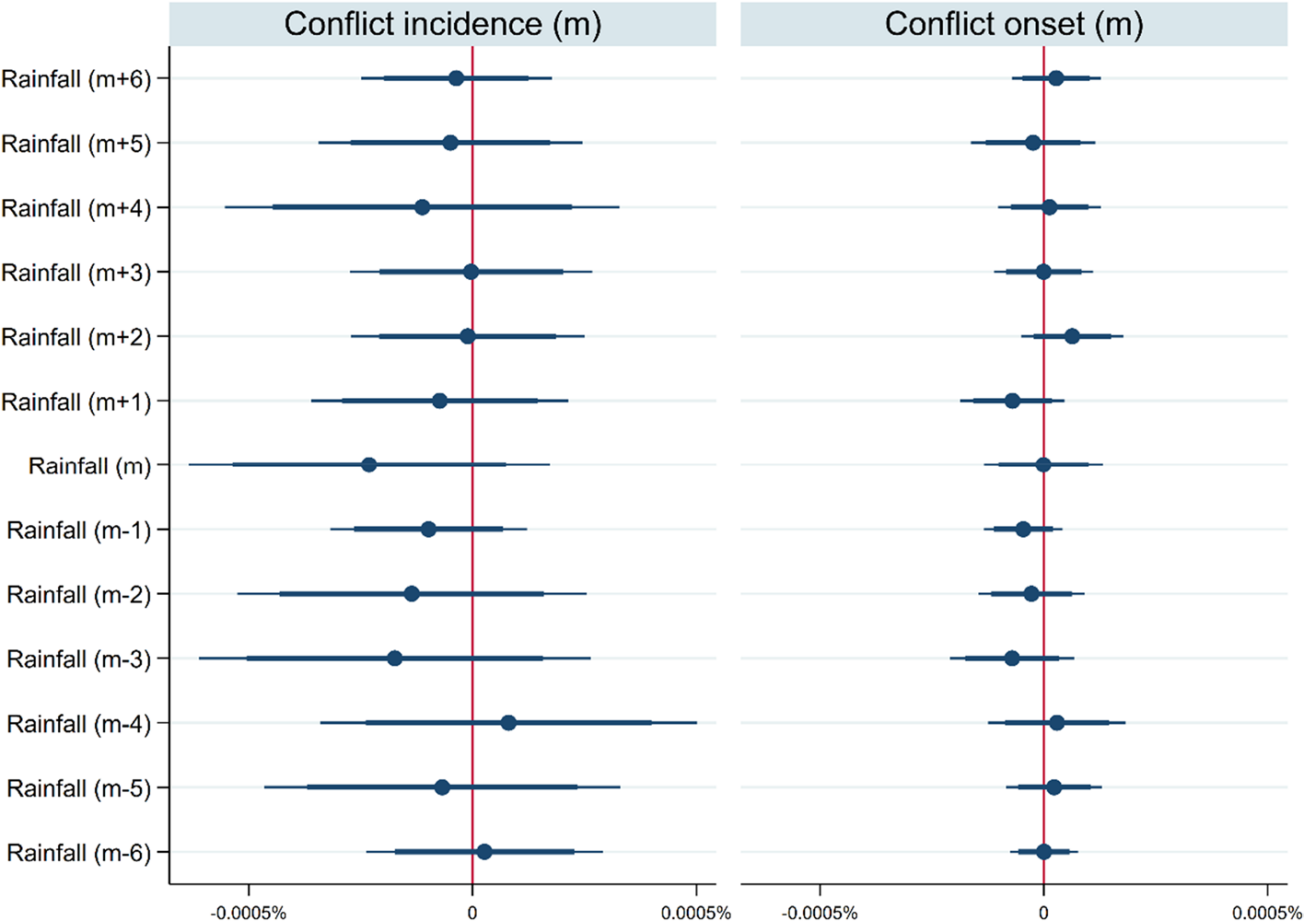
Regressions with six months leads and lags values of rainfall. Each panel of this graph displays the coefficient estimates of a regression at the monthly level when the variable on the top is the dependent variable. The independent variables, that are the values of rainfall in different months, are presented on the left. Each index in parenthesis shows the month when a given variable is accounted for relative to the dependent variable. Blue circles represent coefficient estimates, and the bold and light whiskers around these circles are the 95% and 99% confidence intervals, respectively.

After assuring the robustness of these findings, the explanation for the statistical significance difference between monthly and annual analyses was studied. We propose that this divergence is due to (1) the timing of the effect of rainfall on conflicts and (2) the dispersion of monthly rainfall within years. The absence of a statistical link between conflicts and lag and lead observations of monthly rainfall may shed light on the timing of this impact (Fig. 2). Including past six and future six months of rainfall observations in the regression analyses masks the contemporaneous link between rainfall and conflict incidence. As none of the past and future precipitation variables affect the probability of armed violence, one can argue that their aggregation contributes to nullifying the relationship between rainfall and conflicts at the yearly level. Second, we regressed conflict incidence against the standard deviation of rainfall based on monthly observations at the yearly level to assess the effects of intra-annual rainfall dispersion on conflicts (Table 3, Model 19). The data indicate that a large intra-annual rainfall dispersion reduces the likelihood of conflict, but not the average rainfall. This result suggests that conflicts are less likely to occur when rainfall is concentrated within a few wet months in a year.

**Table 3:** Effects of intra-annual rainfall variations on conflicts.

Variables	Model 19—Dispersion of data averaged at the annual level	Model 19—Dispersion of data averaged at the annual level
	Incidence	Onset
Standard deviation of annual rainfall	− 6.46e−05* (3.60e−05)	3.29e−06 (2.28e−05)
Average annual rainfall	− 5.06e−06 (4.16e−05)	− 1.98e−05 (2.58e−05)
Number of years without conflict (t − 1)	− 0.0151*** (0.00181)	− 0.00530*** (0.000326)
Constant	0.251*** (0.0267)	0.0923*** (0.00509)
Observations	79,546	76,980
R-squared	0.379	0.110
Time FE	YES	YES
Region FE	YES	YES
Period	1990–2020	1991–2020
Sample	Full sample	Full sample

Standard errors are clustered at the country level.

***p < 0.01, **p < 0.05, *p < 0.1.

## Discussion

The impact of climatic factors on armed-conflict outbreaks has been extensively investigated^17,46^. Although a large body of literature has suggested that conflict outbreaks have been associated with rainfall variability^11,17,23,24,43,47,48^, other studies contest this link^2,3,25^. The results of this analysis demonstrate that part of the opposition on this topic can be attributed to the lack of attention paid to the temporal unit of analyses. They also suggest that the inability to find a statistical link between rainfall variability and conflicts at the yearly level can be explained by the disregard of the timing in this relationship and intra-annual rainfall variations.

Most literature surveys agree that compared with the impact of temperature on conflicts, the impact of rainfall is smaller, if not inexistent. For example, studies using averages of climatic factors over six months^48^ and over 37 years as climate indices^49^ show that temperature variations affect conflicts, but not rainfall changes. These findings contribute to researchers’ focus on the risk of conflict caused by temperature variations or indicators of water scarcity such as drought indices instead of rainfall levels^3^. Our analysis suggests that the association between rainfall variability and conflicts may be indiscernible over a large temporal frame. When assessing this relationship, studies must describe further the processes by which rainfall affects conflicts, particularly those that operate at the monthly level^8,49^.

Regarding the specificity of the rainfall’s impact, it appears that this effect has a small magnitude. This result concurs with previous studies suggesting that although climatic variables play a role in conflict outbreaks worldwide, their effects are minor^2,48^. Additionally, results show that monthly rainfall decreases the probability of conflict incidence but not conflict onset: the impact of rainfall primarily concerns conflict-prone regions. Furthermore, the impact of rainfall variability on conflict does not change relative to the national and subnational levels of economic development. This estimate diverges from findings suggesting that the impact of rainfall on conflict is concentrated in poor regions^42,43^. We believe our results provide a pertinent perspective on this issue since we use an estimate of income in monetary terms while previous studies use satellite night light data and infant mortality as indices of economic development. Finally, the impact of rainfall on conflict is scattered over the Americas, Africa, and Asia. Overall, these findings reveal that rainfall has a marginal effect on conflicts in conflict-prone regions, regardless of income level and the continent.

Nevertheless, it is important to reaffirm that this study focuses on the contemporaneous impact of rainfall on conflicts. Previous research focused on how past rainfalls affect armed conflicts by lowering the opportunity costs to enter conflicts after economic activity distortion. Although lag monthly rainfall observations are not linked to conflicts in Fig. 2, we do not negate this conceptual framework but nuance it in two respects.

First, further data explorations highlight that precipitation of the month before a conflict incidence lowers the probability of conflict outbreaks when lead observations are excluded from the model (SI Table S22, Model 20 and 21). Moreover, at the annual level of analysis, the lack of contemporaneous rainfall impact on conflicts contrasts with the effects of lagged rainfall variability that occurred after the three-year mark. After three years, previous yearly rainfall observations are linked to a lower probability of conflict incidence and onset (SI Table S23). This lag effect at the yearly level concurs with previous studies’ conceptual framework. For example, the onset of the Syrian war was preceded by seven consecutive years of drought, causing decreases in income and revolts in the north of the country^50^. However, there were no significant rainfall anomalies at the beginning of civil unrest. Overall, the impact of rainfall on conflicts that operate contemporaneously and near contemporaneously at the monthly level and the lag impact at the yearly level imply that rainfall variability may act as a shock for conflict outbreaks in the short-term (monthly horizon) but also contribute to premeditation of the tradeoff of engaging in conflicts in the long term (yearly horizon).

Second, previous analyses show that local rainfall scarcity in a month increases the probability of social unrest in Africa through its effects on food price shocks^34^ and food insecurity^36^. Another study, using Twitter data in Kenya, highlights that populations can react as fast as the daily level when facing water shortages^51^. However, our results do not fully support this channel whereby rainfall scarcities increase the risk of conflicts at the monthly level. Table 3 suggests that large differences in intra-yearly precipitations decrease conflict risks. It implies that the concentration of rainfall in few months decreases the risks of violence in conflict-prone regions, even if several months have low precipitation. In simple terms, while rainfall scarcity does not increase the likelihood of conflict, large rainfall decreases this likelihood. This interpretation may also explain why state conflicts are less likely during wet months (SI Table S12, Model 16). We conjecture that heavy rainfall may disrupt ongoing armed conflicts. This result may reflect practical difficulties to engage in conflicts concerning flooded roads, reduced visions, or flooded shelter during highly rainy periods, alternatively conflict-actors may shift their focus to rain-fed activities. We believe this interpretation is sensible since this impact is contemporaneous and, thereby, depicts few premeditated actions. Nevertheless, this interpretation is based on the effect of differences in intra-yearly precipitations on conflict risks that is statistically significant only at the 10% level. Further research is required to rigorously investigate this interpretation and the other linkages between rainfall and conflicts.

Indeed, the scope of this study is limited to examining the average effect of rainfall on conflicts at different temporal scales. Although the results of the analysis advocate for a better description of channels through which rainfall affects conflicts at the monthly level, this study does not empirically identify them. Identifying these channels is beyond the scope of this analysis since their regional and contextual specificities require meticulous assessment. Although numerous studies attempt to quantify some of these channels in a global setting, their results are contingent upon rainfall affecting conflicts only through the individual channel they considered in their respective analyses^2,8,25^. This assumption is likely to fail since several experts in the field agree that climate affects conflicts via several channels that are often difficult to quantify^2^. Although complex, rigorous identification strategies show that these channels can be identified through in-depth analyses on channels such as soil quality^52^, dam locations^10^, or seasonal migrations^16^.

Furthermore, we recognized that the coefficients and the statistical significances of rainfall’s effect on the regressions’ conflict outbreaks are much smaller than previous studies’ estimates. This small magnitude may suggest that using the subnational regions as the unit of analysis may not capture all nuances of the impact of rainfall on conflict. Therefore, further research is required to verify our estimates’ reliability at different spatial scales, such as high-resolution pixels or ethnic groups.

Finally, results for African regions show that rainfall levels condition conflicts at the yearly and monthly levels. This may highlight that as Africa is the continent with the highest dependency on rainfall for their economic activities^53,54^, and African countries are highly vulnerable to both rainfall variations and conflicts, yearly deviations in rainfall may significantly affect these regions^41^. Since these results are significant at the 10 percent statistical confidence level, further analyses are required to assess the robustness of this effect.

## Methods

### Data

All data sources are described in Table S2, and their summary statistics at the first order of national administrative units are reported in Table S3. Data on conflict events are derived from UCDP GED version v.21. This data records every armed conflict involving state and non-state actors that led to at least 25 battle-related deaths during at least one calendar year from 1989 to 2020 worldwide^7^. We used UCDP GED non-state conflicts, defined as the confrontation between two organized armed groups, neither of which is the state’s government. The focus on non-state conflicts follows methodologies of previous research tackling the relationship between rainfall and conflicts at the subnational level^11,42,43^. We do not distinguish between internationalized and non-internationalized conflicts. For sensitivity checks of the results, we also use the Armed Conflict Location & Event Data Project (ACLED) data of armed clashes with at least one fatality. The ACLED data reports all political violence and protest events worldwide with their geo-localizations. Since their recording is not based on the 25 battle-related death threshold in their counts, ACLED data primarily differs from UCDP GED data by reporting conflicts rather than armed conflicts. Thus, robustness checks only use ACLED’s armed clashes with at least one fatality for a proportional comparison with UCDP data on armed conflicts.

Using geo-localization information, all conflict events are merged with subnational regions where they happened and then grouped by year and year-months. Regions are the highest administrative unit within a country, as indicated by the Database of Global Administrative Areas (GADM). This administrative unit corresponds to, for example, states in the United States, provinces in China, and prefectures in Japan. Then, with this region-month and region-year data, we create two binary dependent variables: the conflict incidence and onset^42^. The conflict incidence is assigned the value of 1 for observation where at least one conflict occurs within a region during the period of study (month or year) and 0 otherwise. On the other hand, the conflict onset variable is coded 1 if a region experiences a conflict, and if, for at least two consecutive years preceding this conflict, there were none. When these two conditions are not satisfied, we coded this variable as 0. Analyses defining conflict onset as a conflict occurring after five consecutive years of peace yield the same results (see SI Table S20). Due to the conflict onset definition, we cannot distinguish between conflict incidence and onset in the first two years of our sample. Therefore, regressions with conflict onset as the dependent variable only use observations from 1991 to 2020.

Rainfall information is collected from the climate hazards center infrared precipitation with stations (CHIRPS), available at a 0.05 resolution monthly and annually^44^. This rainfall estimate is generated based on global rainfall climatology, satellite-based rainfall estimates, and in situ rainfall observations. Socioeconomic data on annual levels of income per capita, life expectancy, and population stem from the subnational human development index (SDHI)^55^ at the subnational level and the World Bank at the national level. Since SDHI maps use regional boundaries different from those of GADM (the analysis unit), we estimate the centroids of GADM regions and overlay them on the SDHI maps to merge the data.

### Regression analyses

Our preferred model specification uses the statistical method of a review of the literature^3^. It is a panel data fixed effects that compare populations of each region to themselves at different moments in time when they are exposed to different levels of rainfall. This method uses each region as a contrafactual to itself when affected by different levels of rainfall. The result of this comparison can be considered a credible estimate for the causal effect of rainfall on conflicts since we evaluate how each population responds to different rainfall conditions exogenously determined by the climate system. The panel data fixed effects model can be expressed as follows:

1 $${Conflict}_{it}= \beta {Rainfall}_{it} +\gamma {NP}_{i t-1}+{\mu }_{i}+ {\theta }_{t}+ {\varepsilon }_{it}$$

where for region $i$ at the period $t$, ${Conflict}_{it}$ stands as a binary variable of conflict occurrence, ${Rainfall}_{it}$ is the average rainfall. The variable ${NP}_{i t-1}$ is the number of years in peace of each region lagged of one period. It is set to control serial correlation in the model as regions historically conflict-prone are more likely to face conflicts in the future. We assume that regions not experiencing a conflict in the first year of the sample availability (1989) had their first peaceful year. It follows that the year 1989 does not have an estimate of the variable ${NP}_{i t-1}$, and is thereby dropped from the regressions.

The fixed effect of each region is represented by ${\mu }_{i}$ and captures unobserved time-invariant differences across regions such as geography, historical institution, or culture. The term ${\theta }_{t}$ is a dummy variable of the period studied accounted for as years or group year-month depending on the temporal level of analysis. This variable is set to account for monthly average precipitation and seasonality as well as time-trending global patterns like contemporaneous worldwide economic and population growth correlated to rainfall and conflicts. Furthermore, as the ability to report conflicts improves due to Information and Communications Technology (ICT), the variable ${\theta }_{t}$ is crucial for differentiating the PRIO-GED’s growing ability to record conflicts over time from changing global rainfall trends. Finally, $\beta$ is the average impact of rainfall on conflicts, $\gamma$ is the effect of the number of years in peace, and ${\varepsilon }_{it}$ is the idiosyncratic error of region $i$ at the period $t$.

To identify $\beta$, Eq. (1) excludes socio-economic control variables because they may bias the effect of rainfall on conflict^3^. These biases can be summarized into two categories. First, including income or population variables as controls in Eq. (1) attenuates the total effect of rainfall on conflicts as rainfall may affect conflicts through these variables. This issue is referred to as the inclusion of bad control variables. Explicitly, rainfall was shown to cause conflicts via its impact on several factors, such as income shocks (these effects are summarized in literature reviews^2,25^). Thus, including these factors as control variables would produce estimates of the rainfall-conflict linkage when these factors are constant and disregard channels whereby rainfall affects conflicts. For instance, holding income constant by using it as a control variable will disregard the fact that rainfall can affect conflicts through income shocks.

Second, in addition to being affected by rainfall, control variables like income and population may not be directly correlated to conflicts but through unquantifiable features like institutions. In other words, unlike rainfall, these control variables are not exogenous. They are likely correlated with the idiosyncratic error term. The inclusion of bad controls would violate the exogeneity assumption, as some of the independent variables would be correlated with the error term. The violation of the exogeneity assumption results in biased coefficients^56^ (see further details in^3^).

Equation (1) does not imply that rainfall only directly impacts conflicts. As rainfall variations are exogenous, Eq. (1) assumes that the impact of rainfall on conflicts can be captured by $\beta$ regardless of the mechanism underpinning this relationship. Populations in regions are affected by varying levels of rainfall that they cannot predetermine, and their reactions to these variations, via any kind of channel, may result in conflicts.

Conceptually, the analyses use the fixed effects estimation technique to account for time-invariant factors associated with conflicts and precipitation, such as topographies, long-term institutions, cultures, and international boundaries. Nevertheless, we carried out a Hausman test to determine the relevance of this technique. The Hausman test assesses the difference in coefficient estimates between the fixed and the random-effects models and recommends using a fixed-effects model when there is a difference in coefficient. In this analysis, the Hausman test showed the need for the fixed effects technique in models with or without controls (see SI Tables S15–S22). Nonetheless, random effects regressions were also performed to assess the robustness of the fixed effects’ results. A multi-level mixed effects regression was also used as a robustness check to assess the reliability of the interpretations.

The regressions use an OLS technique to concur with previous studies despite the binary nature of the independent variable^3^. This choice resides in the efficiency of OLS at accounting for fixed effects and the ease of interpretation of the coefficients of linear models^52,57^. The impact of rainfall anomalies on conflict outbreaks is interpreted as percentage points by multiplying $\beta$ by 100 since the dependent variables representing conflict outbreaks range between 0 and 1. We also estimate the effects of rainfall on conflicts with a logistic regression model for robustness checks^42^.

Then, we estimate the effects of lagged and lead observations of monthly rainfall variables on conflicts using Eq. (1). This estimation aims to ensure that the results are not spurious and to understand why the effects of rainfall on conflicts may differ by the temporal unit of analysis. Future observations of monthly rainfall should not affect conflict outbreaks of the past. Therefore, the estimated coefficients of future rainfall variables should be statistically indistinguishable from zero, highlighting the robustness of our interpretations of the coefficient of interest. However, prior monthly rainfall variations may cause future conflicts. Hence, if lagged observations of monthly rainfall variability affect conflicts, aggregation of the observations at the annual level may help capture this effect. Yet, if lagged monthly rainfall does not affect conflicts, aggregations at the annual level incorporates some noise in the relationship between rainfall and conflicts.

Despite the large cross-sectional units (Number of regions = 2559), we suspect that serial correlation can be an issue owing to the rainfall’s seasonality. Regressions with block-bootstrap standard errors with 500 repetitions, including clusters of the error terms at the national and regional levels, are used in additional analyses to account for serial correlation across countries and regions. Previous studies show the robustness of this technique against serial correlation^58,59^. Furthermore, rainfall patterns and conflicts are not random in space. Spatial autocorrelation can occur since conflicts in a region can spill over to neighboring regions within the same country. Thus, unless otherwise specified, standard errors are clustered at the country level (161 clusters).

Furthermore, a spatial autoregressive model (SAR) was regressed to account for spatial autocorrelation beyond national borders (see SI Figs. S1 and S2). The SAR assumes that the probability of conflicts in region $i$ at period $t$ is affected by the probability of conflicts in regions with whom it shares a border. The SAR can be expressed as follows:

2 $${Conflict}_{it}= \beta {Rainfall}_{it} +\gamma {NP}_{i t-1}+\rho \sum_{j}^{n}{w}_{ij}{Conflict}_{jt}+ {u}_{it}$$

3 $${u}_{it}= \lambda \sum_{j}^{n}{w}_{ij}{v}_{it}+{ \varepsilon }_{it}$$

4 $${w}_{ij}= \left\{\begin{array}{ll}1 &\quad if\,\, i\,\, and\,\, j\,\, share\,\, border\\ 0& \quad if \,\,i \,\,and\,\, j\,\, do\,\, not\,\, share\,\, a\,\, border\\ 0& \quad if\,\, j=i\end{array}\right.$$

where ${Conflict}_{jt}$ denotes a binary variable of conflict occurrence in region $j$ at period $t$. ${w}_{ij}$ is the element of a contiguity weight matrix ${\varvec{W}}$ of size $n$ × $n$ describing the spatial relationship between the regions $i$ and $j$ (where $n$ is the number of observations in each period). The term ${w}_{ij}$ equals 1 if regions $i$ and $j$ share a geographical boundary and 0 otherwise. The error term ${u}_{it}$ is assumed to comprise a spatially correlated component ${v}_{it}$ and a component ${\varepsilon }_{it}$ that is assumed to be normally distributed across observations in the sample. The term $\rho$ is the spatial autoregressive coefficient describing the influence of neighboring conflicts on local conflicts and $\lambda$ is the autoregressive coefficient describing the correlation of the error terms across neighboring regions. SAR is regressed via the maximum likelihood estimation technique and therefore does not describe a causal estimate^60^. The SAR model excludes ${\mu }_{i}$ and ${\theta }_{t}$ because it fails to converge otherwise on the Software Stata 17.

Finally, another set of robustness checks is performed. An alternative model specification assesses whether the omission of control variables influences the interpretations of our findings. Regions’ average population count, life expectancy, and income per capita are used for this purpose. Additionally, we assess whether changing the measuring unit of rainfall to standardized values over regional long-term rainfall means or logarithm affects the interpretations. We also perform analyses with the number of conflicts per region as the dependent variable since it may better portray the intensity of the effects of rainfall on conflicts. With this dependent variable, the model is regressed with a negative binomial regression technique to account for the left-skewed distribution of count variables^33^. Finally, in other variants of the definition of conflicts is altered. One definition uses UCDP GED data of conflicts involving states. Another definition of conflict consists of changing data sources from UCDP GED to ACLED since they report different types of information. Only armed clashes reported by ACLED are considered.

## Supplementary Information

Supplementary Information.

## Data Availability

Code to replicate all results is available at https://github.com/ThierryCoul/Rainfall_conflcits.
