# Supplementary material for: Identifying the impact of rainfall variability on conflicts at the monthly level
Source: Sci Rep. 2022 Oct 28;12:18162. doi: 10.1038/s41598-022-23079-y (PMC9616923; doi:10.1038/s41598-022-23079-y)
Supplement: Supplementary file 1 — Supplementary Information. [file 41598_2022_23079_MOESM1_ESM.pdf]

Supplementary Information for:

Identifying the impact of rainfall variability on conflicts at the monthly level

This document provides supplementary information for the publication “Identifying the impact of rainfall variability on conflicts at the monthly level.” The document is structured as follows:

- Supplementary text on regression for Figure 1 on page 2.
- Supplementary Figures S1-S3 on pages 3-5.
- Supplementary Tables S1-S32 on pages 6-33.

References herein refer to those listed in the main manuscript.

Supplementary text on regression for Figure 1

We perform regressions of a variant of the model described in Equation (1) for each region that experienced at least one conflict. Hence, these regressions exclude country fixed effects. These regressions are used to assess how the impact of rainfall on conflict varies worldwide. For this purpose, iterations of a reduced form of Equation (1) are performed on data from every single region taken separately. This model can be expressed as follows:

$$Conflict_t = \beta Rainfall_t + \gamma NP_{t-1} + \varphi_y + \varphi_m + \varepsilon_t \qquad \text{(S1)}$$

In Equation (S1), we replace the time fixed effects of the group year-month with two disjoint terms of time fixed effects of years and month denoted by φ_y and φ_m, respectively. This is because data by regions only vary with respect to time (thus, regressions cannot include k dummy variables such that k=n, with n being the number of observations). The terms φ_y and φ_m are included to distinguish the impact of rainfall on conflict from the seasonality of rainfalls. Region-specific $\hat{\beta}$ from Equation (S1) are useful to assess the relationship between rainfall and conflicts per region. However, the results of this regression provide less efficient estimations (larger standard errors) of the impact of rainfall conflicts because of the limited size of the dataset during each iteration.

Figure S1: Number of conflicts per subnational region in the world

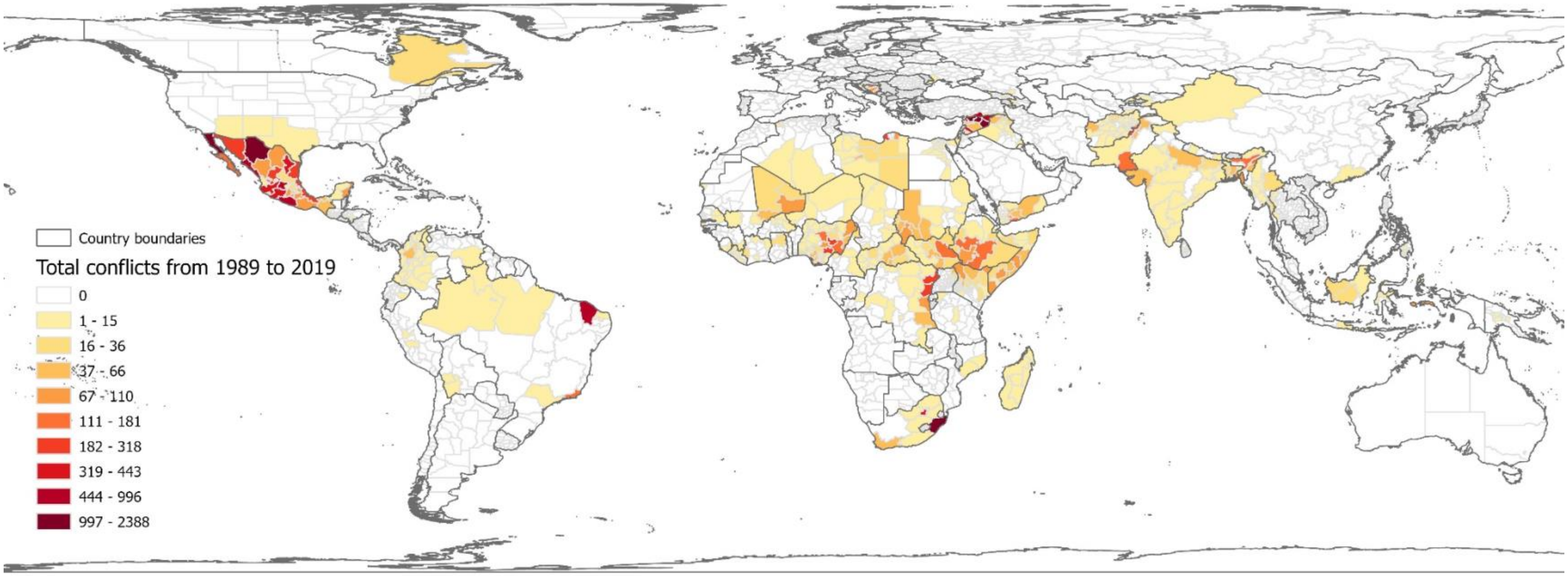

Note: The legend for the total number of conflicts uses the natural breaks of the data for each group. The map is projected using an equal area projection. These maps were created using ArcGIS Pro 2.8 (https://www.esrij.com/products/arcgis-pro/)

Figure S2: Overlap of conflict incidence and first-level administrative boundaries as described by the GADM dataset

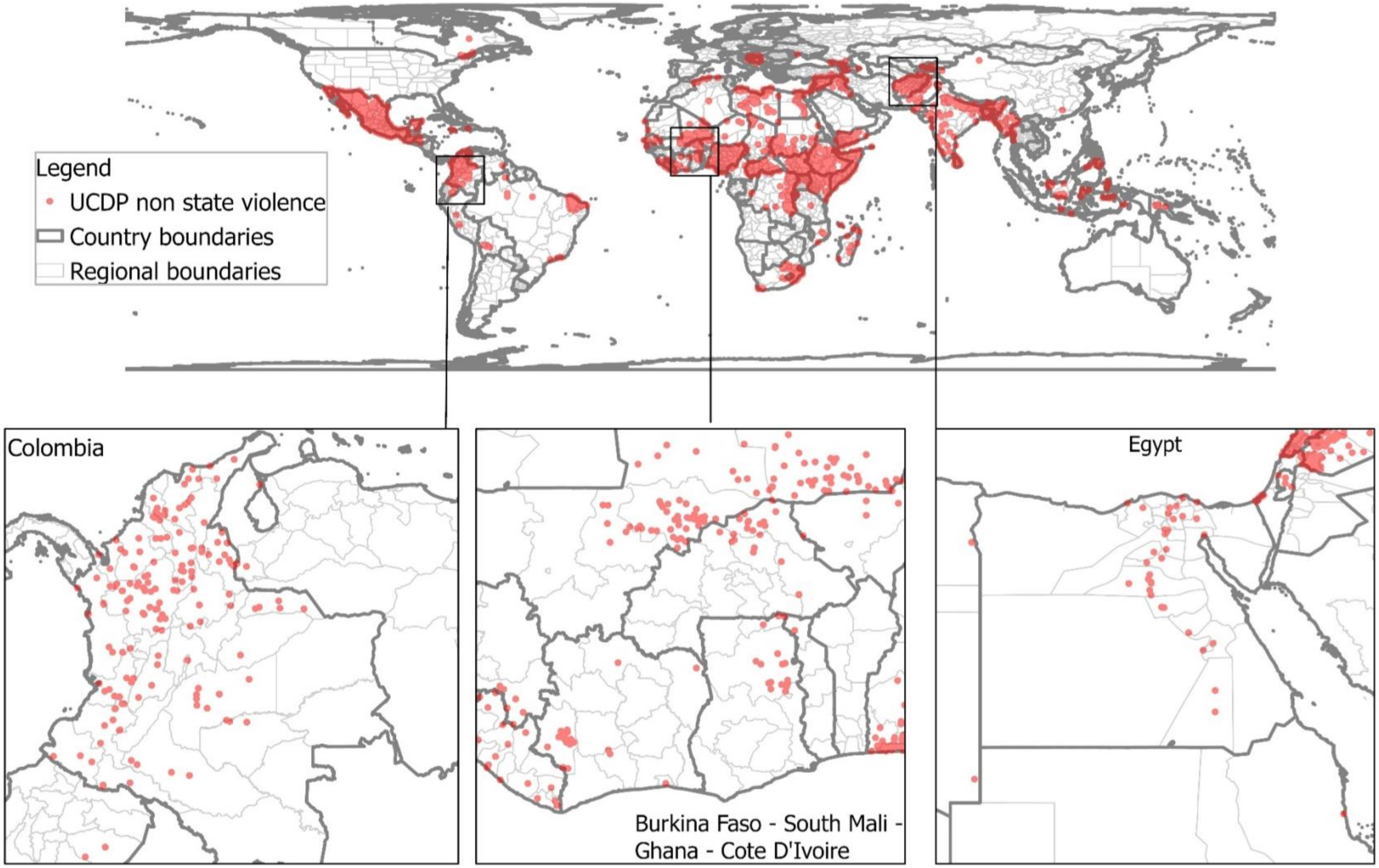

Note: Each dot represents a record of a non-state violent event. The map is projected using an equal area projection. These maps were created using ArcGIS Pro 2.8 (https://www.esrij.com/products/arcgis-pro/)

Figure S3: Regions where control variables are available

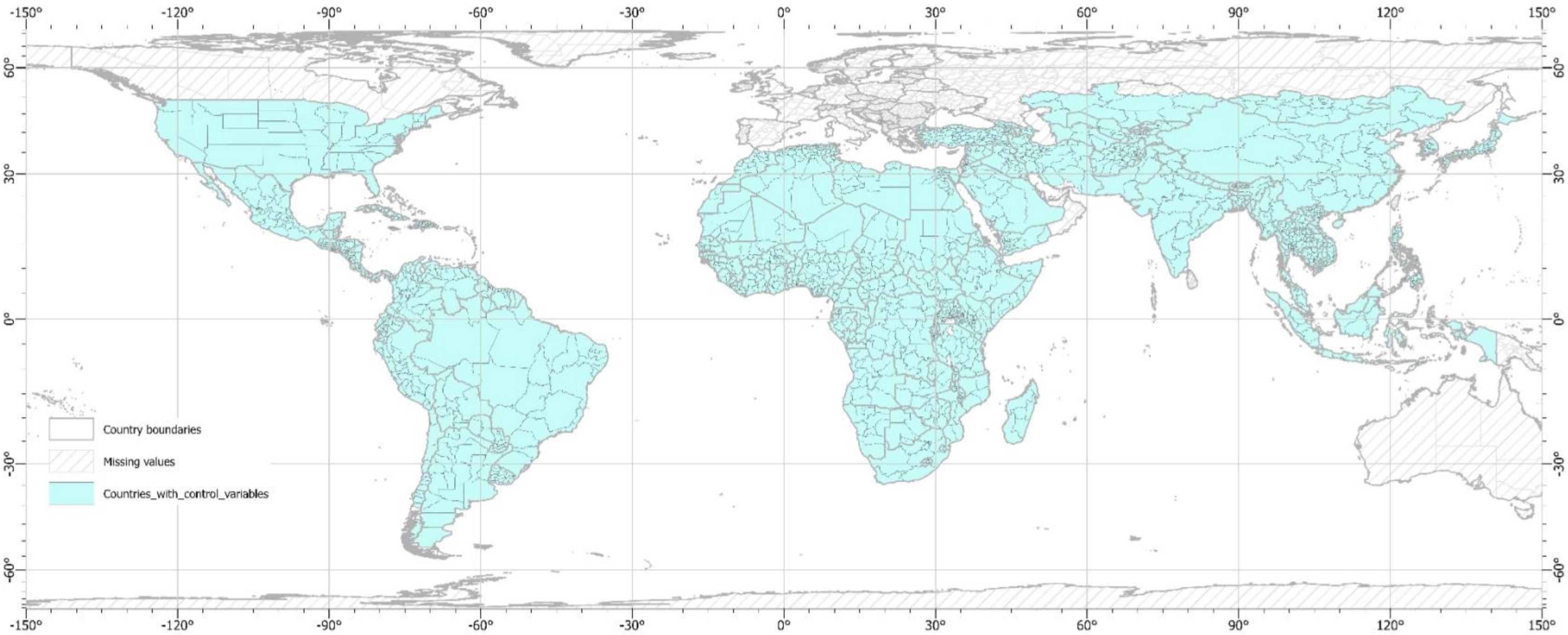

Note: The map is projected using an equal area projection. These maps were created using ArcGIS Pro 2.8 (https://www.esrij.com/products/arcgis-pro/)

<u>Table S1</u>: Heterogeneous effects with respect to income

	Model 2: Monthly level regressions				Model 2: Annual level regressions			
	Heterogeneous effect with respect to regional income		Heterogeneous effect with respect to national income		Heterogeneous effect with respect to regional income		Heterogeneous effect with respect to national income	
VARIABLES	Incidence	Onset	Incidence	Onset	Incidence	Onset	Incidence	Onset
Rainfall (mm)	-2.22e-05	3.49e-06	1.42e-05	5.00e-06	6.12e-06	2.09e-05	8.48e-05	3.62e-05
	(1.91e-05)	(5.82e-06)	(2.15e-05)	(7.50e-06)	(6.14e-05)	(3.37e-05)	(6.83e-05)	(3.41e-05)
Log(regional income per capita)	-0.0136***	-0.00207**			-0.0515**	-0.0203*		
	(0.00417)	(0.000869)			(0.0208)	(0.0116)		
Rainfall * Log(regional income per capita)	2.18e-06	-5.04e-07			-5.42e-07	-2.16e-06		
	(2.31e-06)	(6.41e-07)			(6.99e-06)	(3.75e-06)		
Log (national income per capita)			-0.00406**	0.000343***	-0.00624***	0.00392***	-0.00543**	0.00373***
			(0.00159)	(4.70e-05)	(0.00235)	(0.000512)	(0.00270)	(0.000466)
Rainfall * Log (national income per capita)			-0.0157	-0.00120			-0.0363	-0.00659
			(0.0106)	(0.000793)			(0.0229)	(0.00896)
Constant	0.180***	0.0141*	0.212*	0.00660			-9.95e-06	-4.25e-06
	(0.0456)	(0.00762)	(0.114)	(0.00720)			(7.43e-06)	(3.60e-06)
Observations	739,020	716,532	598,932	584,472	61,585	59,711	50,636	49,431
R-squared	0.146	0.009	0.161	0.010	0.316	0.089	0.306	0.091
Time FE	YES	YES	YES	YES	YES	YES	YES	YES
Region FE	YES	YES	YES	YES	YES	YES	YES	YES
Period	1990 - 2020	1991 - 2020	1990 - 2020	1991 - 2020	1990 - 2020	1991 - 2020	1990 - 2020	1991 - 2020
Sample	Sample where regional income variable is available	Sample where regional income variable is available	Sample where national income variable is available	Sample where national income variable is available	Sample where regional income variable is available	Sample where regional income variable is available	Sample where national income variable is available	Sample where national income variable is available

Note: Standard errors of estimated coefficients are two-way clustered for grouped year-month and country levels. *** p<0.01, ** p<0.05, * p<0.1.

Table S2: Data sources

Variable	Dataset
Conflict outbreaks	Uppsala Conflict Data Program Georeferenced Event Dataset (UCDP GED)
Conflict outbreaks ACLED	Armed Conflict Location and Event Data (ACLED)
Rainfall observations	Climate Hazards Center InfraRed Precipitation with Station data (CHIRPS)
Income per capita	subnational human development index (SDHI)
Population	subnational human development index (SDHI)
Life expectancy	subnational human development index (SDHI)

Table S3: Summary statistics

	Variables	N	Mean	Standard deviation	Minimum	Maximum
Monthly levels statistics	Conflict incidence	985,344	0.00761	0.0869	0	1
	Conflict onset	923,760	0.001090	0.0329988	0	1
	Average rainfall (mm/month)	985,344	103.2	117.1	0	2,119
	Number of years in peace	985,344	14.33	9.442	0	32.00
Yearly levels statistics	Conflict incidence	82,112	0.0316	0.175	0	1
	Conflict onset	76,980	0.012016	0.1089581	0	1
	Total rainfall (mm/year)	82,112	1,238	862.2	0.0201	6,781
	Standard deviation rainfall based on monthly values	82,112	78.56332	56.11629	.0020934	587.5482
	Number of years in peace	82,112	14.66	9.551	0	32
	Regional Income per capita (2013 USD)	61,585	10,044	12,952	360.5	206,429
	National income per capita (2013 USD)	49,911	12,295.24	13,460.58	416.0064	103519.1
	Population (head)	61,285	5,048	10,561	0.798	214,487
	Life expectancy (years)	61,585	66.80	9.240	24.51	85.01
	Logarithm of regional income per capita	61,585	8.643	1.082	5.887	12.24
	Logarithm of national income per capita	49,911	8.892147	1.064871	6.030701	11.54751
	Logarithm of population	61,285	7.467	1.575	-0.226	12.28
	Logarithm of life expectancy	61,585	4.191	0.149	3.199	4.443

Table S4: Regressions with alternative model specifications at the monthly level

	Model 3– Regressions with no time fixed effects		Model 4 – Regressions with random effects		Model 5– Regressions with control variables		Model 6 – Regions with at least one conflict incidence/onset		Model 7 – African regions	
VARIABLES	Incidence	Onset	Incidence	Onset	Incidence	Onset	Incidence	Onset	Incidence	Onset
Rainfall (mm)	-2.36e-06*	-1.31e-06**	-1.29e-05**	-1.44e-06**	-3.74e-06**	-7.72e-07	-1.79e-05***	-4.04e-06	-6.00e-06**	-1.72e-06
	(1.33e-06)	(6.51e-07)	(5.51e-06)	(6.11e-07)	(1.79e-06)	(7.60e-07)	(6.31e-06)	(2.93e-06)	(2.99e-06)	(1.38e-06)
Number of peaceful years (t -1)	-0.000379***	5.89e-05***	-0.00109***	-6.85e-05***	-0.00374***	0.000341***	-0.00580***	0.000603***	-0.00301***	0.000277***
	(0.000120)	(1.35e-05)	(0.000271)	(1.48e-05)	(0.00121)	(4.98e-05)	(0.00129)	(5.50e-05)	(0.000613)	(4.44e-05)
Log (income per capita)					-0.0134***	-0.00213**				
					(0.00396)	(0.000881)				
Log(population)					-0.00264	0.000461				
					(0.00371)	(0.000463)				
Log (life expectancy)					-0.00999	0.00175				
					(0.0333)	(0.00255)				
Constant	0.0133***	0.000334*	0.0246***	0.00228***	0.240	0.00377	0.0834***	0.000839*	0.0508***	-0.00197***
	(0.00176)	(0.000181)	(0.00598)	(0.000402)	(0.172)	(0.0134)	(0.0104)	(0.000454)	(0.00831)	(0.000609)
Observations	982,778	923,760	982,778	923,760	735,420	713,052	207,969	189,000	319,422	300,240
R-squared	0.114	0.007	0.014	0.000	0.146	0.009	0.132	0.007	0.111	0.009
Year FE	NO	NO	NO	NO	YES	YES	YES	YES	YES	YES
Region FE	YES	YES	NO	NO	YES	YES	YES	YES	YES	YES
Period	1990 - 2020	1991 - 2020	1990 - 2020	1991 - 2020	1990 - 2019	1991 - 2019	1990 - 2020	1991 - 2020	1990 - 2020	1991 - 2020
Sample	Full sample	Full sample	Full sample	Full sample	Sample where control variables are available (Figure S3)	Sample where control variables are available (Figure S3)	Sample with at least one conflict incidence (Figure S1)	Sample with at least one conflict incidence (Figure S1)	Sample located in Africa	Sample located in Africa

Note: Standard errors of estimated coefficients are clustered at the country level. *** p<0.01, ** p<0.05, * p<0.1

Table S5: Regressions with alternative model specifications at the yearly level

	Model 3– Regressions with no time fixed effects		Model 4 – Regressions with random effects		Model 5– Regressions with control variables		Model 6 – Regions with at least one conflict incidence/onset		Model 7 – African regions	
VARIABLES	Incidence	Onset	Incidence	Onset	Incidence	Onset	Incidence	Onset	Incidence	Onset
Rainfall (mm)	5.16e-07	-2.68e-06	-6.23e-06	-1.86e-06	1.42e-06	1.89e-06	-3.39e-05	1.94e-06	-2.15e-05*	-1.27e-05**
	(2.48e-06)	(2.01e-06)	(4.21e-06)	(1.60e-06)	(3.96e-06)	(2.58e-06)	(2.60e-05)	(1.69e-05)	(1.22e-05)	(6.24e-06)
Number of peaceful years (t -1)	-0.000592***	0.000701***	-0.00391***	-0.000683***	-0.00626***	0.00394***	-0.00946***	0.00705***	-0.00737***	0.00338***
	(0.000180)	(0.000154)	(0.000746)	(0.000158)	(0.00237)	(0.000519)	(0.00200)	(0.000578)	(0.00182)	(0.000431)
Log (income per capita)					-0.0525***	-0.0227**				
					(0.0162)	(0.00961)				
Log(population)					-0.00308	0.00586				
					(0.0100)	(0.00519)				
Log (life expectancy)					0.00500	0.0170				
					(0.0816)	(0.0286)				
Constant	0.0398***	0.00505*	0.0956***	0.0244***	0.575	0.0349	0.261***	0.000376	0.169***	-0.0144**
	(0.00362)	(0.00301)	(0.0173)	(0.00441)	(0.404)	(0.149)	(0.0353)	(0.0186)	(0.0266)	(0.00579)
Observations	79,546	76,980	79,546	76,980	61,285	59,421	16,833	15,990	25,854	25,020
R-squared	0.275	0.072	0.044	0.004	0.316	0.089	0.211	0.055	0.315	0.081
Year FE	NO	NO	NO	NO	YES	YES	YES	YES	YES	YES
Region FE	YES	YES	NO	NO	YES	YES	YES	YES	YES	YES
Period	1990 - 2020	1991 - 2020	1990 - 2020	1991 - 2020	1990 - 2019	1991 - 2019	1990 - 2020	1991 - 2020	1990 - 2020	1991 - 2020
Sample	Full sample	Full sample	Full sample	Full sample	Sample where control variables are available (Figure S3)	Sample where control variables are available (Figure S3)	Sample with at least one conflict incidence (Figure S1)	Sample with at least one conflict onset (Figure S1)	Sample located in Africa	Sample located in Africa

Note: Standard errors of estimated coefficients are two-way clustered at the country level. *** p<0.01, ** p<0.05, * p<0.1

<u>Table S6</u>: Regressions with alternative regression techniques at the monthly level

	Model 8 – Negative binomial regression	Model 9 - Logit	Model 9 - Logit	Model 10 - Hierarchical linear model	Model 10 - Hierarchical linear model	Model 11 – SAR (see Table S7 for effects)	Model 11 – SAR (see Table S7 for effects)	Model 12 – Block bootstrap	Model 12 – Block bootstrap
VARIABLES	Number of conflicts	Incidence	Onset	Incidence	Onset	Incidence	Onset	Incidence	Onset
Rainfall (mm)	-0.00133***	-0.000704***	-0.000779	-3.78e-06***	-1.22e-06***	-8.44e-06**	-6.75e-06***	-2.98e-06**	-7.16e-07
	(0.000355)	(0.000196)	(0.000543)	(9.86e-07)	(4.00e-07)	(3.42e-06)	(2.10e-06)	(1.46e-06)	(5.57e-07)
Number of peaceful years (t - 1)	-0.203***	-0.260***	0.162***	-0.00129***	8.22e-06	-0.00317 ***	0.00058***	-0.00433***	0.000154***
	(0.0285)	(0.0273)	(0.0138)	(1.45e-05)	(5.78e-06)	(0.00006)	(0.000026)	(0.00120)	(2.35e-05)
Logarithm of income per capita				-0.00186***	-0.000873***				
				(0.000301)	(0.000112)				
Logarithm of population				0.00338***	0.000332***				
				(0.000136)	(5.12e-05)				
Logarithm of life expectancy				0.118***	0.00205***				
				(0.00180)	(0.000697)				
W. Dependent variable						0.43423 ***	-0.0714***		
						(0.00558)	(0.000138)		
W. Error						0.167***	0.0724***		
						(0.000347)	(0.000138)		
Constant	-5.406***	-7.005***	-9.248***	-0.473***	-0.00226			0.00530***	-0.00269***
	(1.087)	(0.406)	(0.768)	(0.00724)	(0.00261)			(0.00128)	(0.000463)
Observations	982,778	207,586	187,560	735,420	713,052	214,485	200,265	982,778	923,760
Pseudo R-squared / R-squared	0.361	0.323	0.0818			0.0237	0.0010	0.042	0.008
Time FE	YES	YES	YES	NO	NO	NO	NO	YES	YES
Region FE	YES	YES	YES	NO	NO	YES	YES	YES	YES
Period	1990 - 2020	1990 - 2020	1991 - 2020	1990 - 2019	1991 - 2019	1990 - 2020	1991 - 2020	1990 - 2020	1991 - 2020
Sample	Full sample	Sample with at least one conflict incidence (Figure S1)	Sample with at least one conflict onset (Figure S1)	Sample where control variables are available (Figure S3)	Sample where control variables are available (Figure S3)	Sample with at least one conflict incidence (Figure S1)	Sample with at least one conflict onset (Figure S1)	Full sample	Full sample

Note: Clustered standard errors at the country level in parentheses in Models 8, 9, 11, 12, and 13. Robust standard errors are displayed in Model 10. The hierarchical models use random intercepts at the country level, and regressions are performed using a maximum likelihood fit. Logit and SAR models exclude observations with no variation in their dependent variable during the study period because the model failed to converge otherwise. *** p<0.01, ** p<0.05, * p<0.1

Table S7: Direct, indirect, and total effects of rainfall on conflicts at the monthly level based on SAR, Model 11.

VARIABLES	Incidence	Onset
Direct effect	-9.01e-06**	-6.7e-06***
	(3.65e-06)	(2.11e-06)
Indirect effect	-5.40e-06**	-4.27e-07**
	(2.19e-06)	(1.65e-07)
Total effect	-0.0000144**	-6.34e-06***
	(5.83e-06)	(1.97e-06)

Note: *** p<0.01, ** p<0.05, * p<0.1. Delta-Method of estimation of the standard errors in parentheses

<u>Table S8:</u> Regressions with alternative regression techniques at the yearly level

	Model 8 – Negative binomial regression	Model 9 - Logit	Model 9 - Logit	Model 10 - Hierarchical linear model	Model 10 - Hierarchical linear model	Model 11 – SAR (see Table S9 for effects)	Model 11 – SAR (see Table S9 for effects)	Model 12 – Block bootstrap	Model 12 – Block bootstrap
VARIABLES	Number of conflicts	Incidence	Onset	Incidence	Onset	Incidence	Onset	Incidence	Onset
Rainfall (mm)	-0.000199	-0.000383	-0.000197	-1.91e-06	-4.68e-07	-9.32e-07	-1.96e-05	-3.01e-06	-8.11e-07
	(0.000327)	(0.000272)	(0.000351)	(1.45e-06)	(9.44e-07)	(9.96e-06)	(1.27e-05)	(3.62e-06)	(5.84e-07)
Number of peaceful years (t -1)	-0.0485***	-0.0860***	0.174***	-0.00356***	0.000192***	-0.00316***	0.008092***	-0.00614***	0.000200***
	(0.0133)	(0.0138)	(0.0196)	(9.64e-05)	(6.47e-05)	(0.00031)	0.0003175	(0.00187)	(2.41e-05)
Logarithm of income per capita				-0.0131***	-0.00933***				
				(0.00198)	(0.00124)				
Logarithm of population				0.0126***	0.00340***				
				(0.000896)	(0.000572)				
Logarithm of life expectancy				0.327***	0.0160**				
				(0.0119)	(0.00779)				
W. Dependent variable						0.5794325***	-0.3062697***		
						(.0123507)	(0.0208035)		
W. Error						0.285***	0.211***		
						(0.00204)	(0.00148)		
Constant	-2.067***			-1.252***	-0.000486			0.0315***	0.00653***
	(0.577)			(0.0464)	(0.0291)			(0.00805)	(0.00241)
Observations	79,546	16,554	15,480	61,285	59,421	17,376	16,833	82,112	78,780
Pseudo R-squared / R-squared	0.341					0.0417	0.0052	0.031	0.015
Time FE	YES	YES	YES	NO	NO	NO	NO	YES	YES
Region FE	YES	YES	YES	NO	NO	YES	YES	YES	YES
Period	1990 - 2020	1990 - 2020	1991 - 2020	1990 - 2019	1991 - 2019	1990 - 2020	1991 - 2020	1990 - 2020	1990 - 2020
Sample	Full sample	Sample with at least one conflict incidence (Figure S1)	Sample with at least one conflict onset (Figure S1)	Sample where control variables are available (Figure S3)	Sample where control variables are available (Figure S3)	Sample with at least one conflict incidence (Figure S1)	Sample with at least one conflict onset (Figure S1)	Full sample	Full sample

Note: Clustered standard errors at the country level in parentheses in Models 8, 9, 11, 12, and 13. Robust standard errors are displayed in Model 10. The hierarchical models use random intercepts at the country level, and regressions are performed using a maximum likelihood fit. Logit and SAR models exclude observations with no variation in their dependent variable during the study period because the model failed to converge otherwise. *** p<0.01, ** p<0.05, * p<0.1

Table S9: Direct, Indirect, and total effects of rainfall on conflicts at the yearly level based on SAR, Model 11.

VARIABLES	Incidence	Onset
Direct effect	-1.06e-06	-0.0000202
	(0.0000114)	(0.0000131)
Indirect effect	-1.05e-06	4.76e-06
	(0.0000112)	(3.10e-06)
Total effect	-2.11e-06	-.0000154
	(0.000022)	(9.97e-06)

Note: *** p<0.01, ** p<0.05, * p<0.1. Delta-Method of estimation of the standard errors in parentheses

Table S10: Regressions with alternative definitions of the independent variable at the monthly level

	Model 13 – Standardized values of rainfall by regional average	Model 13- Standardized values of rainfall by regional average	Model 14- Standardized values of rainfall by regional average and restriction to observation where: $-3 \leq z \leq 3$	Model 14- Standardized values of rainfall by regional average and restriction to observation where: $-3 \leq z \leq 3$	Model 15 – Logarithm of rainfall	Model 15 - Logarithm of rainfall
VARIABLES	Incidence	Onset	Incidence	Onset	Incidence	Onset
Rainfall standardized	-0.000398***	-7.52e-05	-0.000386***	-7.90e-05		
	(0.000139)	(5.31e-05)	(0.000136)	(5.68e-05)		
Log (rainfall (mm))					-0.000214***	-5.32e-05
					(7.82e-05)	(3.62e-05)
Number of peaceful years (t-1)	-0.00433***	0.000256***	-0.00435***	0.000254S***	-0.00433***	0.000256***
	(0.00119)	(3.02e-05)	(0.00120)	(3.04e-05)	(0.00119)	(3.02e-05)
Constant						
Observations	982,778	923,760	972,958	914,353	982,778	923,760
R-squared	0.150	0.008	0.150	0.008	0.150	0.008
Time FE	YES	YES	YES	YES	YES	YES
Region FE	YES	YES	YES	YES	YES	YES
Period	1990 - 2019	1991 - 2019	1990 - 2019	1991 - 2019	1990 - 2019	1991 - 2019
Sample	Full sample	Full sample	Observations whose rainfall standardized value is not lower or higher than 3	Observations whose rainfall standardized value is not lower or higher than 3	Full sample	Full sample

Note: Standard errors of estimated coefficients are clustered at the country levels. We used cutoffs for outliers of -3 and 3 based on visual checks with histogram and boxplot distributions in Model 14. Using cutoffs of -1 and 1 yields coefficients similar to those presented in Model 14. *** p<0.01, ** p<0.05, * p<0.1

Table S11: Regressions with alternative definitions of the independent variable at the yearly level

	Model 13 – Standardized values of rainfall by regional average	Model 13- Standardized values of rainfall by regional average	Model 14- Standardized values of rainfall by regional average and restriction to observation where: $-3 \leq z \leq 3$	Model 14- Standardized values of rainfall by regional average and restriction to observation where: $-3 \leq z \leq 3$	Model 15 – Logarithm of rainfall	Model 15 - Logarithm of rainfall
VARIABLES	Incidence	Onset	Incidence	Onset	Incidence	Onset
Rainfall standardized	-0.000730	0.000304	-0.000754	0.000361		
	(0.00111)	(0.000547)	(0.00111)	(0.000530)		
Log (rainfall (mm))					-0.00534	0.00226
					(0.00536)	(0.00284)
Number of peaceful years (t-1)	-0.00614***	0.00303***	-0.00614***	0.00307***	-0.00614***	0.00303***
	(0.00185)	(0.000315)	(0.00187)	(0.000322)	(0.00185)	(0.000315)
Constant	0.121***	-0.0342***	0.121***	-0.0347***	0.157***	-0.0494**
	(0.0271)	(0.00475)	(0.0273)	(0.00487)	(0.0544)	(0.0194)
Observations	79,546	76,980	79,254	76,701	79,546	76,980
R-squared	0.288	0.080	0.288	0.080	0.288	0.080
Time FE	YES	YES	YES	YES	YES	YES
Region FE	YES	YES	YES	YES	YES	YES
Period	1990 - 2019	1991 - 2019	1990 - 2019	1991 - 2019	1990 - 2019	1991 - 2019
Sample	Full sample	Full sample	Observations whose rainfall standardized value is not lower or higher than 3	Observations whose rainfall standardized value is not lower or higher than 3	Full sample	Full sample

Note: Standard errors of estimated coefficients are clustered at the country levels. We used cutoffs for outliers of -3 and 3 based on visual checks with histogram and boxplot distributions in Model 14. Using cutoffs of -1 and 1 yields coefficients similar to those presented in Model 14. *** p<0.01, ** p<0.05, * p<0.1

Table S12: Regressions with alternative data/conflict definition at the monthly level

	Model 16 – State conflicts	Model 16 - State conflicts	Model 17 - ACLED armed clashes	Model 17 - ACLED armed clashes	Model 18 - country level	Model 18 - country level
VARIABLES	Incidence	Onset	Incidence	Onset	Incidence	Onset
Rainfall (mm)	-1.00e-05**	7.79e-08	-6.93e-06***	-6.07e-06***	-3.93e-06***	-8.23e-07
	(5.02e-06)	(7.12e-07)	(2.41e-06)	(2.15e-06)	(1.44e-06)	(7.11e-07)
Number of peaceful years (t-1)	-0.00545***	-4.28e-06	-0.00898***	-0.000726	-0.00152***	-6.46e-05***
	(0.00162)	(3.64e-05)	(0.00145)	(0.000556)	(6.23e-05)	(1.60e-05)
Constant	0.114***	0.00216***	0.177***	0.0300***	0.0249***	0.00180***
	(0.0234)	(0.000552)	(0.0245)	(0.00984)	(0.000832)	(0.000237)
Observations	982,778	923,760	677,424	615,840	65,876	61,920
R-squared	0.272	0.007	0.225	0.123	0.238	0.034
Time FE	YES	YES	YES	YES	YES	YES
Region FE	YES	YES	YES	YES		
Country FE					YES	YES
Period	1990 - 2020	1991 - 2020	1997 - 2018	1999 - 2018	1990 - 2020	1991 - 2020
Sample	Full sample	Full sample	Full sample	Full sample	Full sample	Full sample

Note: Standard errors of estimated coefficients are clustered at the country level. *** p<0.01, ** p<0.05, * p<0.1

Table S13: Regressions with alternative data/conflict definition at the yearly level

	Model 16 – State conflicts	Model 16 - State conflicts	Model 17 - ACLED armed clashes	Model 17 - ACLED armed clashes	Model 18 - country level	Model 18 - country level
VARIABLES	Incidence	Onset	Incidence	Onset	Incidence	Onset
Rainfall (mm)	3.18e-06	3.86e-06	-7.89e-06*	-6.74e-06	-2.49e-08	-1.71e-07
	(5.93e-06)	(2.51e-06)	(4.64e-06)	(4.54e-06)	(5.45e-06)	(9.99e-07)
Number of peaceful years (t-1)	-0.00747***	7.27e-05	-0.00725***	0.00295**	-0.00335***	-1.27e-05
	(0.00165)	(0.000388)	(0.00118)	(0.00117)	(0.000245)	(4.55e-05)
Constant	0.209***	0.0164**	0.214***	0.0166	0.0669***	0.000547
	(0.0250)	(0.00672)	(0.0204)	(0.0209)	(0.00711)	(0.00131)
Observations	79,546	76,980	61,584	56,452	5,332	5,160
R-squared	0.462	0.066	0.369	0.258	0.425	0.039
Time FE	YES	YES	YES	YES	YES	YES
Region FE	YES	YES	YES	YES		
Country FE					YES	YES
Period	1990 - 2020	1991 - 2020	1998 - 2018	1999 - 2018	1990 - 2020	1991 - 2020
Sample	Full sample	Full sample	Full sample	Full sample	Full sample	Full sample

Note: Standard errors of estimated coefficients are clustered at the country level. *** p<0.01, ** p<0.05, * p<0.1

Table S14: Regressions with the same sample of different model specifications at the monthly level (1)

	Model 1 - Fixed effects	Model 1 - Fixed effects	Model 3 - Fixed effects without time fixed effects	Model 3 - Fixed effects without time fixed effects	Model 4 - Random effects	Model 4 - Random effects	Model 5 - with control variables	Model 5 - with control variables
VARIABLES	Incidence	Onset	Incidence	Onset	Incidence	Onset	Incidence	Onset
Mean precipitation	-1.99e-05**	-4.15e-06	-1.46e-05*	-6.38e-06*	-5.04e-05*	-6.07e-07	-5.41e-05**	-1.63e-06
	(8.55e-06)	(3.70e-06)	(8.06e-06)	(3.48e-06)	(2.52e-05)	(2.19e-06)	(2.61e-05)	(2.27e-06)
Number of peaceful years (t-1)	-0.00515***	0.000602***	-0.00349***	0.000534***	-0.00439***	0.000215***	-0.00449***	0.000229***
	(0.00137)	(4.52e-05)	(0.000974)	(5.81e-05)	(0.00114)	(5.02e-05)	(0.00119)	(4.96e-05)
Log (income per capita)							-0.00145	-0.00142***
							(0.00743)	(0.000460)
Log(population)							0.00283	0.000456**
							(0.00374)	(0.000226)
Log (life expectancy)							0.0932**	0.00620
							(0.0426)	(0.00396)
Constant	0.0778***	0.000263	0.0634***	0.00103**	0.0741***	0.00321***	-0.322*	-0.0143
	(0.0119)	(0.000450)	(0.00843)	(0.000453)	(0.0182)	(0.000759)	(0.170)	(0.0135)
Observations	134,328	134,328	134,328	134,328	134,328	134,328	134,328	134,328
R-squared	0.128	0.009	0.102	0.004	0.031	0.000	0.037	0.001
Time FE	YES	YES	NO	NO	NO	NO	YES	YES
Region FE	YES	YES	YES	YES	NO	NO	YES	YES
Period	1991-2019							
Sample	Sample that experiences at least one conflict and where control variables are available							

Note: Standard errors of estimated coefficients are clustered at the country level. *** p<0.01, ** p<0.05, * p<0.1

Table S15: Regressions with the same sample of different model specifications at the yearly level (1)

	Model 1 - Fixed effects	Model 1 - Fixed effects	Model 3 - Fixed effects without time fixed effects	Model 3 - Fixed effects without time fixed effects	Model 4 - Random effects	Model 4 - Random effects	Model 5 - with control variables	Model 5 - with control variables
VARIABLES	Incidence	Onset	Incidence	Onset	Incidence	Onset	Incidence	Onset
Mean precipitation	-1.61e-05	7.57e-06	9.83e-06	-5.57e-06	-9.88e-06	6.49e-06	-1.48e-05	4.65e-06
	(3.67e-05)	(2.38e-05)	(2.53e-05)	(1.99e-05)	(1.10e-05)	(4.19e-06)	(1.19e-05)	(3.44e-06)
Number of peaceful years (t-1)	-0.00798***	0.00726***	-0.00448***	0.00642***	-0.0103***	0.00298***	-0.0103***	0.00310***
	(0.00256)	(0.000473)	(0.00161)	(0.000607)	(0.00186)	(0.000540)	(0.00196)	(0.000532)
Log (income per capita)							-0.0281*	-0.0137**
							(0.0155)	(0.00522)
Log(population)							0.0111	0.00450*
							(0.00895)	(0.00247)
Log (life expectancy)							0.300***	0.0547
							(0.0977)	(0.0455)
Constant	0.219***	-0.0119	0.164***	0.00884	0.232***	0.0239***	-0.862**	-0.123
	(0.0471)	(0.0251)	(0.0285)	(0.0208)	(0.0354)	(0.00886)	(0.364)	(0.160)
Observations	11,194	11,194	11,194	11,194	11,194	11,194	11,194	11,194
R-squared	0.194	0.059	0.164	0.051	0.044	0.009	0.054	0.011
Year FE	YES	YES	NO	NO	NO	NO	YES	YES
Region FE	YES	YES	YES	YES	NO	NO	YES	YES
Period	1991-2019							
Sample	Sample that experiences at least one conflict and where control variables are available							

Note: Standard errors of estimated coefficients are clustered at the country level. *** p<0.01, ** p<0.05, * p<0.1

Table S16: Regressions with the same sample of different model specifications at the monthly level (2)

	Model 8 - Negative binomial	Model 9 - Logit	Model 9 - Logit	Model 10 - Hierarchical model	Model 10 - Hierarchical model	Model 11 – SAR (see Table S14 for effects)	Model 11 – SAR (see Table S14 for effects)	Model 12 - Block bootstrap	Model 12- Block bootstrap
VARIABLES	Number of conflicts	Incidence	Onset	Incidence	Onset	Incidence	Onset	Incidence	Onset
Mean precipitation	-0.000514	-0.000842***	-0.000732	-2.58e-05***	-3.77e-06*	-8.63e-06**	-6.22e-06**	-1.99e-05**	-4.15e-06
	(0.000413)	(0.000307)	(0.000665)	(5.04e-06)	(1.95e-06)	(4.05e-06)	(2.61e-06)	(8.43e-06)	(3.71e-06)
Number of peaceful years (t-1)	-0.185***	-0.229***	0.170***	-0.00474***	0.000293***	-0.00254***	0.000643***	-0.00515***	0.000602***
	(0.0328)	(0.0286)	(0.0150)	(7.19e-05)	(2.85e-05)	(6.44e-05)	(3.48e-05)	(0.00137)	(4.51e-05)
Log (income per capita)				-0.0203***	-0.00224***				
				(0.00133)	(0.000403)				
Log(population)				0.00656***	0.000534**				
				(0.000608)	(0.000210)				
Log (life expectancy)				0.274***	0.00649**				
				(0.00742)	(0.00259)				
W. Dependent variable						-0.3036657***	0.20991***		
						(0.007857)	(0.013789)		
W. Error						0.157***	0.0694***		
						(0.000410)	(0.000176)		
Constant	-3.851***	-4.610***	-5.494***	-0.950***	-0.0101			0.0310***	0.00686
	(0.546)	(0.528)	(0.525)	(0.0280)	(0.00897)			(0.0102)	(0.00430)
Observations	134,328	134,328	134,328	134,328	134,328	134,328	134,328	134,328	134,328
R-squared	0.231	0.322	0.107			0.0252	0.0006	0.044	0.007
Time FE	YES	YES	YES	NO	NO	NO	NO	YES	YES
Region FE	YES	YES	YES	NO	NO	NO	NO	YES	YES
Period	1991-2019								
Sample	Sample that experiences at least one conflict and where control variables are available								

Note: Clustered standard errors at the country level in parentheses in Models 8, 9, 11, 12, and 13. Robust standard errors are displayed in Model 10. The hierarchical models use random intercepts at the country level, and regressions are performed using a maximum likelihood fit. *** p<0.01, ** p<0.05, * p<0.1

Table S17: Direct, Indirect, and total effects of rainfall on conflicts at the yearly level based on SAR, Model 11, Table S14

VARIABLES	Incidence	Onset
Direct effect	-9.33e-06**	-6.27e-06**
	(4.37e-06)	(2.63e-06)
Indirect effect	-5.85e-06**	7.85e-07**
	(2.74e-06)	(3.38e-07)
Total effect	-0.0000152**	-5.48e-06**
	(7.11e-06)	(2.30e-06)

Note: *** p<0.01, ** p<0.05, * p<0.1. Delta-Method of estimation of the standard errors in parentheses

Table S18: Regressions with the same sample of different model specifications at the yearly level (2)

	Model 8 - Negative binomial	Model 9 - Logit	Model 9 - Logit	Model 10 - Hierarchical model	Model 10 - Hierarchical model	Model 11 – SAR (see Table S18 for effects)	Model 11 – SAR (see Table S18 for effects)	Model 12 - Block bootstrap	Model 12- Block bootstrap
VARIABLES	Number of conflicts	Incidence	Onset	Incidence	Onset	Incidence	Onset	Incidence	Onset
Mean precipitation	-0.000186	-0.000207	0.000118	-1.78e-05***	4.56e-06	1.71e-06	-1.32e-05	-1.61e-05	7.57e-06
	(0.000456)	(0.000416)	(0.000447)	(6.24e-06)	(3.58e-06)	(1.22e-05)	(1.55e-05)	(3.59e-05)	(2.32e-05)
Number of peaceful years (t-1)	-0.0292*	-0.0700***	0.207***	-0.0101***	0.00410***	-0.00286***	0.00831***	-0.00798***	0.00726***
	(0.0163)	(0.0183)	(0.0223)	(0.000470)	(0.000346)	(0.000371)	(0.000383)	(0.00253)	(0.000472)
Log (income per capita)				-0.0829***	-0.0234***				
				(0.00809)	(0.00457)				
Log(population)				0.0193***	0.00630***				
				(0.00385)	(0.00239)				
Log (life expectancy)				0.709***	0.0781**				
				(0.0473)	(0.03251)				
W. Dependent variable						-0.371519	0.493553		
						(0.02126)	(0.016347)		
W. Error						0.276***	0.207***		
						(0.00249)	(0.00182)		
Constant	-2.927***	-3.483***	-6.764***	-2.187***	-0.161			0.105***	0.0108
	(0.608)	(0.401)	(0.626)	(0.171)	(0.114)			(0.0395)	(0.0247)
Observations	11,194	11,194	11,194	11,194	11,049	11,194	11,194	11,194	11,194
R-squared	0.147	0.206	0.151			0.0294	0.0091	0.042	0.038
Time FE	YES	YES	YES	NO	NO	NO	NO	YES	YES
Region FE	YES	YES	YES	NO	NO	NO	NO	YES	YES
Period	1991-2019								
Sample	Sample that experiences at least one conflict and where control variables are available								

Note: Clustered standard errors at the country level in parentheses in Models 8, 9, 11, 12, and 13. Robust standard errors are displayed in Model 10. The hierarchical models use random intercepts at the country level, and regressions are performed using a maximum likelihood fit. *** p<0.01, ** p<0.05, * p<0.1

Table S19: Direct, Indirect, and total effects of rainfall on conflicts at the yearly level based on SAR, Model 11, Table S16

VARIABLES	Incidence	Onset
Direct effect	2.00e-06	-0.0000138
	(0.0000142)	(0.0000161)
Indirect effect	2.01e-06	3.69e-06
	(0.0000142)	(4.32e-06)
Total effect	4.00e-06	-0.0000101
	(0.0000284)	(0.0000118)

Note: *** p<0.01, ** p<0.05, * p<0.1. Delta-Method of estimation of the standard errors in parentheses

Table S20: Regressions with the same sample of different model specifications at the monthly level (3)

	Model 13 - Standardized values of rainfall by regional average	Model 13 - Standardized values of rainfall by regional average	Model 15 - log rainfall	Model 15 - log rainfall	Model 16 - State conflicts	Model 16 - State conflicts
VARIABLES	Incidence	Onset	Incidence	Onset	Incidence	Onset
Rainfall standardized	-0.00156**	-0.000213				
	(0.000611)	(0.000266)				
Log (rainfall (mm))			-0.00101***	-0.000241*		
			(0.000247)	(0.000141)		
Rainfall (mm)					-3.86e-05	9.41e-07
					(2.54e-05)	(2.44e-06)
Number of peaceful years (t-1)	-0.00515***	0.000602***	-0.00515***	0.000602***	-0.00233	4.05e-05
	(0.00137)	(4.52e-05)	(0.00137)	(4.52e-05)	(0.00170)	(5.58e-05)
Constant	0.0761***	-0.000103	0.0792***	0.000646	0.142***	0.00404***
	(0.0116)	(0.000381)	(0.0116)	(0.000547)	(0.0133)	(0.000544)
Observations	134,328	134,328	134,328	134,328	134,328	134,328
R-squared	0.128	0.009	0.128	0.009	0.292	0.007
Time FE	YES	YES	YES	YES	YES	YES
Region FE	YES	YES	YES	YES	YES	YES
Period	1991-2019					
Sample	Sample that experiences at least one conflict and where control variables are available					

Note: Standard errors of estimated coefficients are clustered at the country level. *** p<0.01, ** p<0.05, * p<0.1

Table S21: Regressions with the same sample of different model specifications at the yearly level (3)

	Model 13 - Standardized values of rainfall by regional average	Model 13 - Standardized values of rainfall by regional average	Model 15 - log rainfall	Model 15 - log rainfall	Model 16 - State conflicts	Model 16 - State conflicts
VARIABLES	Incidence	Onset	Incidence	Onset	Incidence	Onset
Rainfall standardized	0.00152	0.00426				
	(0.00649)	(0.00373)				
Log (rainfall (mm))			-0.00149	0.0216		
			(0.0354)	(0.0202)		
Rainfall (mm)					4.56e-05	-1.47e-06
					(3.16e-05)	(2.22e-05)
Number of peaceful years (t-1)	-0.00794***	0.00729***	-0.00796***	0.00728***	-0.00351*	0.000562
	(0.00256)	(0.000479)	(0.00256)	(0.000475)	(0.00208)	(0.000688)
Constant	0.202***	-0.00417	0.212	-0.142	0.280***	0.0435*
	(0.0208)	(0.00388)	(0.231)	(0.129)	(0.0375)	(0.0243)
Observations	11,194	11,194	11,194	11,194	11,194	11,194
R-squared	0.193	0.059	0.193	0.059	0.477	0.045
Time FE	YES	YES	YES	YES	YES	YES
Region FE	YES	YES	YES	YES	YES	YES
Period	1991-2019					
Sample	Sample that experiences at least one conflict and where control variables are available					

Note: Standard errors of estimated coefficients are clustered at the country level. *** p<0.01, ** p<0.05, * p<0.1

Table S22: Regressions with lag monthly rainfall observations

	Model 20 – Regressions with t and t-1		Model 21 - Regressions with one lag of rainfall		Model 22 – Regression with t until t-10 rainfall	
VARIABLES	Incidence	Onset	Incidence	Onset	Incidence	Onset
Rainfall	-2.98e-06**	-8.33e-07			-2.85e-06	-8.35e-08
	(1.40e-06)	(6.13e-07)			(1.88e-06)	(5.62e-07)
Rainfall (m-1)			-2.86e-06*	-1.15e-06*	-1.17e-06	-5.48e-07
			(1.57e-06)	(6.10e-07)	(9.56e-07)	(3.95e-07)
Rainfall (m-2)					-1.66e-06	-2.14e-07
					(1.53e-06)	(4.99e-07)
Rainfall (m-3)					-1.99e-06	-7.33e-07
					(1.75e-06)	(5.62e-07)
Rainfall (m-4)					8.05e-07	2.61e-07
					(1.49e-06)	(6.70e-07)
Rainfall (m-5)					-9.96e-07	2.06e-07
					(1.69e-06)	(3.97e-07)
Rainfall (m-6)					-9.08e-07	-6.99e-08
					(1.13e-06)	(2.90e-07)
Rainfall (m-7)					-8.31e-08	3.32e-07
					(9.81e-07)	(4.13e-07)
Rainfall (m-8)					-1.68e-06	-2.09e-07
					(1.60e-06)	(4.61e-07)
Rainfall (m-9)					1.50e-06	4.76e-07
					(1.57e-06)	(3.48e-07)
Rainfall (m-10)					-2.00e-06	-2.27e-07
					(1.40e-06)	(4.79e-07)
Rainfall (m-11)					7.51e-08	-3.99e-07
					(1.18e-06)	(5.18e-07)
Number of peaceful years (t-1)	-0.0043***	0.000256***	-0.00433***	0.000256***	-0.00435***	0.000256***
	(0.00119)	(3.02e-05)	(0.00119)	(3.02e-05)	(0.00121)	(3.02e-05)
Constant	0.0699***	-0.00269***	0.0699***	-0.00266***	0.0726***	-0.00265***
	(0.0171)	(0.000463)	(0.0171)	(0.000462)	(0.0180)	(0.000514)
Observations	982,778	923,760	982,778	923,760	957,118	923,760
R-squared	0.150	0.008	0.150	0.008	0.152	0.008
Time FE	YES	YES	YES	YES	YES	YES
Region FE	YES	YES	YES	YES	YES	YES
Period	1990 - 2020	1991 - 2020	1990 - 2020	1991 - 2020	1990 - 2020	1991 - 2020
Sample	Full sample	Full sample	Full sample	Full sample	Full sample	Full sample

Note: Standard errors of estimated coefficients are clustered at the country level. *** p<0.01, ** p<0.05, * p<0.1

Table S23: Regressions with lag yearly rainfall observations

	Model 20 – Regressions with t and t-1		Model 21 - Regressions with one lag of rainfall		Model 22 – Regression with t until t-10 rainfall	
VARIABLES	Incidence	Onset	Incidence	Onset	Incidence	Onset
Rainfall	-3.32e-06	-1.89e-07			-2.67e-06	8.27e-07
	(3.32e-06)	(2.03e-06)			(3.85e-06)	(2.81e-06)
Rainfall (y-1)	-1.58e-06	2.15e-07	-2.01e-06	1.91e-07	4.09e-07	1.92e-06
	(2.49e-06)	(2.02e-06)	(2.68e-06)	(1.99e-06)	(3.04e-06)	(2.33e-06)
Rainfall (y-2)					-5.81e-07	-2.16e-06
					(3.64e-06)	(2.52e-06)
Rainfall (y-3)					-5.94e-06*	-7.24e-06**
					(3.18e-06)	(2.80e-06)
Rainfall (y-4)					-3.38e-06	-3.24e-06*
					(2.99e-06)	(1.74e-06)
Rainfall (y-5)					-3.84e-06	-2.37e-06
					(3.51e-06)	(2.10e-06)
Rainfall (y-6)					-1.50e-06	-4.50e-06*
					(4.21e-06)	(2.40e-06)
Rainfall (y-7)					-1.28e-05***	-9.68e-06***
					(4.89e-06)	(3.27e-06)
Rainfall (y-8)					-1.09e-05**	-5.86e-06**
					(4.40e-06)	(2.29e-06)
Rainfall (y-9)					-9.70e-06**	-5.24e-06***
					(4.01e-06)	(1.91e-06)
Rainfall (y-10)					-9.68e-06*	-3.26e-06
					(4.92e-06)	(2.26e-06)
Number of peaceful years (y-1)	-0.00782***	0.00304***	-0.00781***	0.00304***	-0.00684***	0.00490***
	(0.00212)	(0.000335)	(0.00212)	(0.000335)	(0.00226)	(0.000530)
Constant	0.149***	-0.0327***	0.146***	-0.0329***	0.235***	-0.0254
	(0.0323)	(0.00622)	(0.0311)	(0.00584)	(0.0521)	(0.0179)
Observations	79,546	76,980	79,546	76,980	56,452	56,452
R-squared	0.304	0.081	0.304	0.081	0.368	0.106
Year FE	YES	YES	YES	YES	YES	YES
Region FE	YES	YES	YES	YES	YES	YES
Period	1990 - 2020	1991 - 2020	1990 - 2020	1991 - 2020	1999 - 2020	1999 - 2020
Sample	Full sample	Full sample	Full sample	Full sample	Full sample	Full sample

Note: Standard errors of estimated coefficients are clustered at country level. *** p<0.01, ** p<0.05, * p<0.1

Table S24: Conflict onset defined with a 5-year cutoff

VARIABLES	Model 1 - Observations averaged at the monthly level Onset (5 years from the previous conflict)	Model 1- Observations averaged at the yearly level Onset (5 years from the previous conflict)
Rainfall (mm)	-9.79e-08	1.32e-06
	(4.06e-07)	(1.33e-06)
Number of peaceful years (t-1)	0.000363***	0.00420***
	(2.57e-05)	(0.000286)
Constant	-0.00523***	-0.0605***
	(0.000420)	(0.00455)
Observations	831,384	69,282
R-squared	0.007	0.074
Time FE	YES	YES
Region FE	YES	YES
Period	1993 - 2020	1993 - 2020
Sample	Full sample	Full sample

Note: Standard errors of estimated coefficients are clustered at the country level. *** p<0.01, ** p<0.05, * p<0.1

Table S25: Hausmann test at the monthly level without control variables for rainfall on conflict incidence

	Coefficients			
	(b)<br>Fixed effect coefficients	(B)<br>Random effect coefficients	(b -B)<br>Difference	sqrt(diag(V_b-V_B))<br>S.E.
Rainfall at the monthly level	-2.92e-06	-3.30e-06	3.80e-07	8.97e-08
Number of years without conflict (t -1)	-.0002943	-.0003196	.0000253	5.17e-07

b = consistent under Ho and Ha; obtained from xtreg (linear regression on panel data in Stata)
B = inconsistent under Ha, efficient under Ho; obtained from xtreg (linear regression on panel data in Stata)

Test: Ho: difference in coefficients not systematic
chi2(1) = (b-B)'[(V_b-V_B)^(-1)](b-B)
= 2418.82
Prob>chi2 = 0.0000

Table S26: Hausmann test at the monthly level with control variables for rainfall on conflict incidence

	Coefficients			
	(b)<br>Fixed effect coefficients	(B)<br>Random effect coefficients	(b -B)<br>Difference	sqrt(diag(V_b-V_B))<br>S.E.
Rainfall at the monthly level	-3.46e-06	-3.93e-06	4.71e-07	1.09e-07
Number of years without conflict (t -1)	-.0001055	-.0000775	-.000028	8.01e-07
Logarithm of income	-.000214	-.0037892	.0035752	.0002447
Logarithm of population	.0279428	.0081225	.0198203	.0005302
Logarithm of income	.0859373	.0918203	-.005883	.0009599

b = consistent under Ho and Ha; obtained from xtreg (linear regression on panel data in Stata)
B = inconsistent under Ha, efficient under Ho; obtained from xtreg (linear regression on panel data in Stata)

Test: Ho: difference in coefficients not systematic
chi2(4) = (b-B)'[(V_b-V_B)^(-1)](b-B)
= 2700.04
Prob>chi2 = 0.0000

Table S27: Hausmann test at the monthly level without control variables for rainfall on conflict onset

	Coefficients			
	(b)<br>Fixed effect coefficients	(B)<br>Random effect coefficients	(b -B)<br>Difference	sqrt(diag(V_b-V_B))<br>S.E.
Rainfall at the monthly level	-2.92e-06	-3.30e-06	3.80e-07	8.97e-08
Number of years without conflict (t -1)	.000055	-.0000291	.0000841	1.17e-06

b = consistent under Ho and Ha; obtained from xtreg (linear regression on panel data in Stata)
B = inconsistent under Ha, efficient under Ho; obtained from xtreg (linear regression on panel data in Stata)

Test: Ho: difference in coefficients not systematic
chi2(1) = (b-B)'[(V_b-V_B)^(-1)](b-B)
= 5212.31
Prob>chi2 = 0.0000

Table S28: Hausmann test at the monthly level without control variables for rainfall on conflict onset

	Coefficients			
	(b)<br>Fixed effect coefficients	(B)<br>Random effect coefficients	(b -B)<br>Difference	sqrt(diag(V_b-V_B))<br>S.E.
Rainfall at the monthly level	-1.22e-06	-1.52e-06	2.99e-07	2.40e-07
Number of years without conflict (t -1)	.0001653	-.0000367	.000202	6.08e-06
Logarithm of income	-.002643	-.0003707	-.0022723	.0001673
Logarithm of population	-.0008275	.000257	-.0010845	.0002476
Logarithm of income	-.0028323	-.000778	-.0020542	.0008013

b = consistent under Ho and Ha; obtained from xtreg (linear regression on panel data in Stata)
B = inconsistent under Ha, efficient under Ho; obtained from xtreg (linear regression on panel data in Stata)

Test: Ho: difference in coefficients not systematic
chi2(4) = (b-B)'[(V_b-V_B)^(-1)](b-B)
= 2700.04
Prob>chi2 = 0.0000

<u>Table S29:</u> Hausmann test at the yearly level without control variables for rainfall on conflict incidence

	Coefficients			
	(b)<br>Fixed effect coefficients	(B)<br>Random effect coefficients	(b -B)<br>Difference	sqrt(diag(V_b-V_B))<br>S.E.
Rainfall at the yearly level	4.16e-07	-5.41e-06	5.82e-06	2.12e-06
Number of years without conflict (t -1)	-0.0005394	-0.0016326	.0010932	0.000016

b = consistent under Ho and Ha; obtained from xtreg (linear regression on panel data in Stata)
B = inconsistent under Ha, efficient under Ho; obtained from xtreg (linear regression on panel data in Stata)

Test: Ho: difference in coefficients not systematic
chi2(1) = (b-B)'[(V_b-V_B)^(-1)](b-B)
= 5209.30
Prob>chi2 = 0.0000

<u>Table S30:</u> Hausmann test at the yearly level with control variables for rainfall on conflict incidence

	Coefficients			
	(b)<br>Fixed effect coefficients	(B)<br>Random effect coefficients	(b -B)<br>Difference	sqrt(diag(V_b-V_B))<br>S.E.
Rainfall at the yearly level	2.64e-06	-5.27e-06	7.91e-06	2.42e-06
Number of years without conflict (t -1)	-.0013313	-.001465	.0001336	.0000845
Logarithm of income	-.030263	-.0215659	-.0086971	.002339
Logarithm of population	.0509697	.0114561	.0395136	.0039153
Logarithm of life expectancy	.1744616	.1245399	.0499217	.0098058

b = consistent under Ho and Ha; obtained from xtreg (linear regression on panel data in Stata)
B = inconsistent under Ha, efficient under Ho; obtained from xtreg (linear regression on panel data in Stata)

Test: Ho: difference in coefficients not systematic
chi2(4) = (b-B)'[(V_b-V_B)^(-1)](b-B)
= 2617.36
Prob>chi2 = 0.0000

<u>Table S31:</u> Hausmann test at the yearly level without control variables for rainfall on conflict onset

	Coefficients			
	(b)<br>Fixed effect coefficients	(B)<br>Random effect coefficients	(b -B)<br>Difference	sqrt(diag(V_b-V_B))<br>S.E.
Rainfall at the yearly level	-2.44e-06	-1.76e-06	-6.74e-07	1.55e-06
Number of years without conflict (t -1)	0.0006337	-0.0006721	0.0013059	0.000018

b = consistent under Ho and Ha; obtained from xtreg (linear regression on panel data in Stata)
B = inconsistent under Ha, efficient under Ho; obtained from xtreg (linear regression on panel data in Stata)

Test: Ho: difference in coefficients not systematic
chi2(1) = (b-B)'[(V_b-V_B)^(-1)](b-B)
= 5440.94
Prob>chi2 = 0.0000

<u>Table S32:</u> Hausmann test at the yearly level without control variables for rainfall on conflict onset

	Coefficients			
	(b)<br>Fixed effect coefficients	(B)<br>Random effect coefficients	(b -B)<br>Difference	sqrt(diag(V_b-V_B))<br>S.E.
Rainfall at the yearly level	1.14e-07	-1.93e-06	2.04e-06	1.98e-06
Peace years (t-1)	.0021144	-.0004922	.0026066	.0000705
Logarithm of income	-.0312493	-.0040443	-.027205	.0019779
Logarithm of population	-.0144895	.0027782	-.0172677	.0029212
Logarithm of life expectancy	-.0441821	-.010461	-.0337211	.0093547

b = consistent under Ho and Ha; obtained from xtreg (linear regression on panel data in Stata)
B = inconsistent under Ha, efficient under Ho; obtained from xtreg (linear regression on panel data in Stata)

Test: Ho: difference in coefficients not systematic
chi2(4) = (b-B)'[(V_b-V_B)^(-1)](b-B)
= 4081.78
Prob>chi2 = 0.0000
